# Supramolecular Recognition of a DNA Four‐Way Junction by an M_2_L_4_ Metallo‐Cage, Inspired by a Simulation‐Guided Design Approach

**DOI:** 10.1002/anie.202504866

**Published:** 2025-05-05

**Authors:** Samuel J. Dettmer, Hugo D. Williams, Richard Napier, Joseph M. Beames, Steffan Walker‑Griffiths, Timothy D. Craggs, Michael J. Hannon

**Affiliations:** ^1^ School of Chemistry University of Birmingham Edgbaston Birmingham B15 2TT UK; ^2^ School of Life Sciences University of Warwick Coventry CV4 7AL UK; ^3^ School of Mathematical and Physical Sciences Centre for Single‐Molecule Biology University of Sheffield Sheffield S3 7HF UK

**Keywords:** Bio‐inorganic, DNA four‐way junctions, DNA‐recognition, Metallo‐cage, Supramolecular chemistry

## Abstract

DNA four‐way junctions (4WJs) play an important biological role in DNA repair and recombination, and viral regulation, and are attractive therapeutic targets. Compounds that recognise the junction structure are rare; in this work, we describe cationic metallo‐supramolecular M_2_L_4_ cages as a new type of 4WJ binder with nanomolar affinities. A combination of molecular dynamics (MD) simulations and biophysical experiments show that the size and shape of a compound comprising square planar Pd or Pt and anthracene‐based ligands is an excellent fit for the 4WJ cavity. Whilst the cage is also capable of binding to three‐way junctions (3WJs) and Y‐fork structures, we show that the 4WJ is the preferred DNA target, and that duplex B‐DNA is not a competitor. Among 3WJs, T‐shape bulged 3WJs are bound more preferably than perfect Y‐shaped 3WJs. Whilst previous work studying M_2_L_4_ metallo‐supramolecular cages has focused on binding inside their structures, this work exploits the external aromatic surfaces of the supramolecule, creating a supramolecular guest that ideally matches the DNA host cavity. This approach allows available structures to be identified as potential junction binders and then screened for their fit to a nucleic acid junction target using simulations. This has potential to significantly accelerate discovery.

## Graphical abstract

DNA Holliday junctions, or four‐way junctions (4WJs) are an attractive therapeutic target due to their prevalence in DNA replication and repair, and viral disease. Combining experimental and computational methods, we show that quadruple‐stranded dinuclear platinum metallo‐cages are an excellent size and shape match for the cavity of the 4WJ open conformation, binding with nanomolar affinity and high selectivity for junctions.

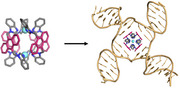

## Introduction

DNA is an important biomolecule responsible for storing the genetic code in many diverse organisms. Compounds that bind to DNA and interrupt its usual processes offer exciting potential as therapeutic agents. Cisplatin and anthracyclines bind the DNA duplex and are major players in the treatment of cancers, but lack specificity and are therefore associated with unwanted side effects.^[^ ^1^, ^2^, ^3^, ^4^ ^]^ An attractive approach to achieve higher selectivity is to look beyond the conventional double helical B‐DNA structure and target noncanonical DNA structures, which are less common but are formed when DNA is processed, and so are attractive as both functional and more exclusive targets.

An important class of higher order nucleic acid structure is the junction, comprising of multiple strands (*n* > 2) that meet at a branchpoint. Holliday junctions, or four‐way junctions (4WJ), are well‐known DNA structures that occur across the genome, not only at inverted repeat sequences, but as vital intermediates in cell replication, unwinding of stalled replication forks and repair of double‐strand breaks. Cancer cells are associated with higher levels of double‐strand breaks, making Holliday junctions an attractive target. The more elusive three‐way junction (3WJ) is implicated in DNA transactions as well as being associated with triplet nucleotide repeat expansion diseases such as Huntington's and fragile X syndrome.^[^ ^5^, ^6^, ^7^, ^8^, ^9^, ^10^, ^11^ ^]^

**Figure 1:**
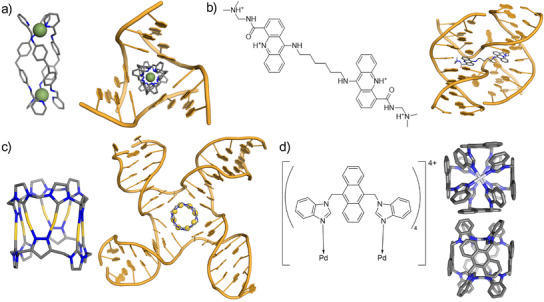
a) 3D structure of an iron triple‐helicate cylinder (*left*) and the crystal structure of the cylinder bound inside the cavity of a 3WJ (PDB 2ET0; *right*).^[^ ^12^ ^]^ b) Chemdraw image of a bis‐acridine bis‐intercalator (*left*) and the crystal structure of this compound bound to a 4WJ in the closed conformation (PDB 2GWA; *right*).^[^ ^23^ ^]^ c) 3D structure of an organometallic Au pillarplex (*left*) and a molecular dynamics snapshot of this compound bound inside the cavity of a 4WJ in the open conformation (*right*).^[^ ^24^ ^]^ d) Chemdraw structure of Zhou and Sun's palladium cage (*left*) and end‐on and side‐on views of the 3D structure this cage (CCDC 1048711; *right*).^[^ ^25^ ^]^ Hydrogens have been omitted for clarity in all images.

Cationic triple‐helicate cylinders are able to bind to DNA and RNA 3WJs by threading through the cavity at the branchpoint of the three strands (Figure 1a).^[^ ^12^, ^13^, ^14^ ^]^ Importantly, these agents possess a shape and size profile that is highly complementary with the 3WJ cavity. Notably, the cylinder presents the planar faces of its aromatic rings (rather than their C─H edges) on its exterior creating aryl surfaces that match and face‐face *π‐*stack with the nucleobase surfaces at the junction branchpoint. Removing access to those surfaces by encapsulating the cylinder within a cucurbit[10]uril macrocycle deactivates the 3WJ binding.^[^ ^15^ ^]^ Monchaud and colleagues have described how the same, and similar, surfaces can be presented by organic azacryptands to also achieve 3WJ binding.^[^ ^16^, ^17^, ^18^ ^]^ Other helicate or mesocate (*meso* analogues of helicates) complexes with aliphatic surfaces or aryl C─H edges have also been described to bind 3WJs,^[^ ^19^, ^20^, ^21^, ^22^ ^]^ though their structures will not match/fill the open cavity as effectively.

More frequently occurring in biology is the four‐way “Holliday” junction (4WJ).^[^ ^26^ ^]^ This junction adopts two major conformations: *open cruciform* and *closed X‐stacked* (Figure 1b,c).^[^ ^26^, ^27^ ^]^ The majority of known, small molecule 4WJ binders target the closed X‐stacked form either by binding across two adjacent X‐stacked arms, or by intercalating between the central stacked base pairs of the closed‐up junction (Figure 1b).^[^ ^23^, ^28^, ^29^, ^30^, ^31^, ^32^ ^]^ However, it is the open cruciform conformation that is observed during homologous recombination and other cellular processes.^[^ ^33^, ^34^ ^]^ 4WJs are also important structures found in viral DNA and RNA genomes,^[^ ^35^, ^36^ ^]^ so open 4WJs are targets for both cancer and antiviral treatments. Small peptides are able to enter and stabilise a *partially open* 4WJ cavity,^[^ ^37^, ^38^ ^]^ and we recently reported that organometallic Au pillarplexes, octanuclear tetracations with convex surfaces, also bind in 4WJ cavities (Figure 1c).^[^ ^24^ ^]^ We reasoned that four flat aryl surfaces arranged to form a square tube will be the ideal shape to bind open 4WJs, and that quadruple‐stranded cages with appropriate size, shape and aromatic ligands should provide this configuration. We report such a system and its high affinity 4WJ binding herein.

Quadruple‐stranded metallo‐cages assembled by square planar palladium(II) are a well‐known class of supramolecular compounds with 3D nanometre‐scale cage geometries. The primary interest in these compounds has stemmed from their well‐defined *interior* cavities, which can act as size‐dependent binding pockets for a wide variety of guests, including anions, cations, fullerenes and cancer drugs.^[^ ^39^, ^40^, ^41^, ^42^, ^43^, ^44^, ^45^, ^46^, ^47^, ^48^ ^]^ Work towards applications of these compounds has seen the development of stimuli‐responsive cages, to allow for control in the uptake and release of cargo held within the cage structure.^[^ ^49^, ^50^, ^51^, ^52^ ^]^ A small number of studies have investigated the uptake and cytotoxicity of metallo‐cages in cells^[^ ^53^, ^54^, ^55^, ^56^, ^57^ ^]^; however, the binding of these compounds to biomolecules is largely unexplored.

From considering the available M_2_L_4_ compounds in literature, we identified a palladium(II) cage prepared from a bis‐benzimidazole ligand containing an anthracene spacer (BIMA) reported by Zhou and Sun.^[^ ^25^ ^]^ The crystal structure reveals our desired square‐tube shape with the four anthracenes presenting their aryl surfaces to the outside of the complex (Figures 1d, S9, S10). As a tetracation (like the cylinders and pillarplexes), it should bind anionic DNA, and anthracenes are an ideal size to stack with a DNA base‐pair. While the pillarplex 4WJ‐binder was a little small for the 4WJ cavity, this BIMA complex is larger and better matched. Therefore, we chose this metallo‐cage to explore whether the *exterior* of such a complex might be used to bind 4WJs in the open cruciform conformation.

## Results and Discussion

### Molecular Dynamics Screening

**Figure 2:**
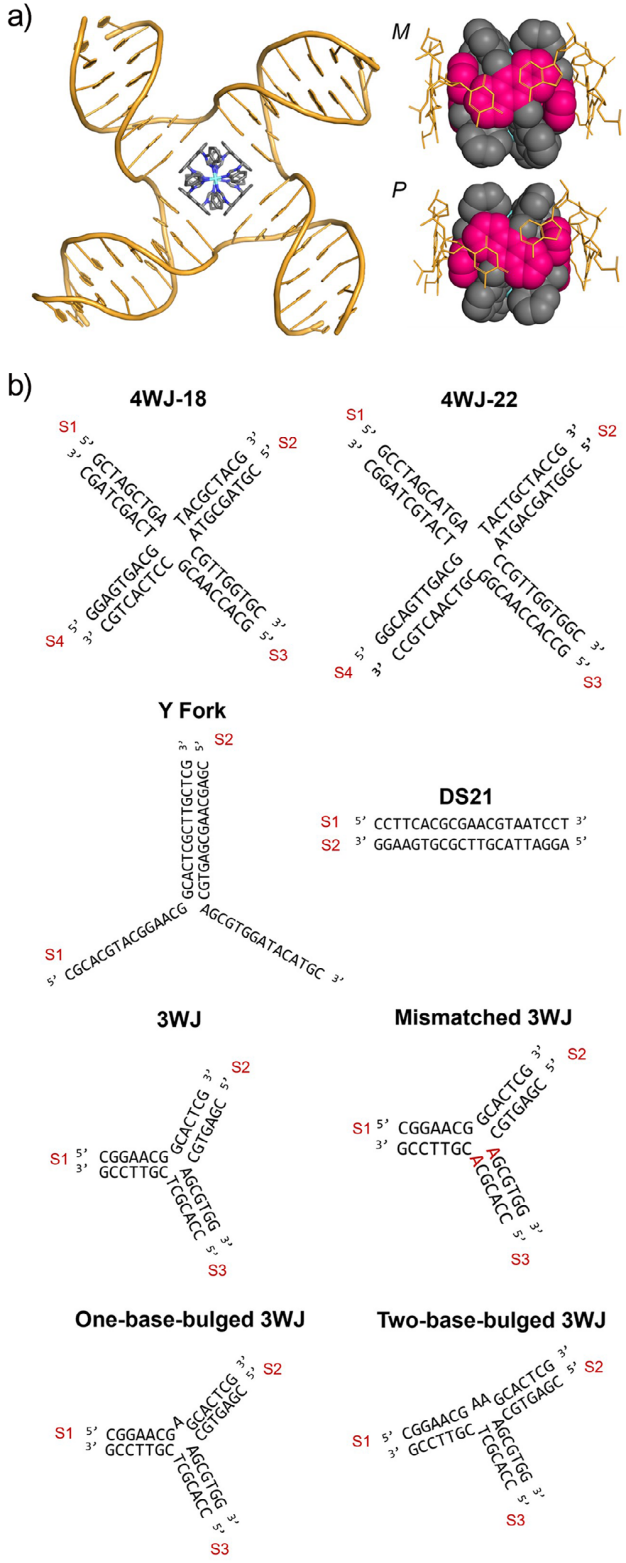
a) MD snapshot of the M enantiomer of Pt‐BIMA bound inside the 4WJ (adapted from PDB 1XNS). PtBIMA remains inside the cavity for the duration of the simulations. Close up images of each enantiomer bound in the 4WJ, where only branchpoint base pairs are shown (orange sticks) and the anthracene units on PtBIMA are shown in pink. b) DNA sequences used in this work.

Molecular dynamics (MD) simulations are valuable to probe and explain experimentally observed effects of DNA‐binding,^[^ ^24^, ^35^ ^]^ but now we use them to predict the potential fit for 4WJ binding before experiment. From the reported crystal structure, both the palladium compound (Pd‐BIMA) and a platinum analogue (Pt‐BIMA) were DFT optimised and parameterised for MD, yielding identical geometry predictions (Figure S11). Like the cylinder helicates, the compound has axial chirality, so both enantiomers (M and P) were considered. As an initial starting point for MD, a snapshot was taken from a simulation we reported previously, in which an organometallic pillarplex is bound in the heart of a 4WJ cavity (adapted from PDB 1XNS) and where all four branchpoint base pairs are intact.^[^ ^37^ ^]^ The pillarplex was removed and Pt‐BIMA then placed inside the open cavity. In simulations with the M enantiomer (3 at 4 µs each), the junction quickly adjusted itself in order to align its branchpoint base pairs with the compound's anthracene surfaces (Figure 2a). The compound remained inside the open cavity and the four anthracene units maintained *π*‐*π* contacts with the four branchpoint base pairs for the duration of the simulations. This is facilitated by the helicity of the M enantiomer, which allows for perfect alignment of the anthracene units parallel to the branchpoint base pairs. The *π‐π* distances between the anthracene unit and the base pairs dynamically fluctuate between 3.3 and 3.9 Å (Figure S12), which is within the expected range for optimal *π‐π* interactions. Conversely, the helicity of the P enantiomer is such that the anthracene units are instead naturally aligned perpendicular to the axis of the branchpoint base pairs (Figure 2a). Nevertheless, the P enantiomer similarly remained inside the 4WJ for the duration of the simulations (3 at 4 µs each), though the 4WJ showed more conformational freedom in these simulations (Figure S13).

Simulations using the partially open branchpoint conformation seen in the PDB 1XNS crystal structure as a starting point were then conducted with PdBIMA and PtBIMA (three per compound per enantiomer at 2 µs each) placed outside of the junction cavity, as opposed to directly inside. Both the PdBIMA and PtBIMA complexes act very similarly in MD, and they quickly enter the junction, after which the cavity arranges into a square shape and establishes all four branchpoint base pairs, equivalent to the structure seen in the earlier simulations. Simulations in which PtBIMA was placed near the *closed* X‐stacked 4WJ structure did not see entry into the heart of the 4WJ; most likely because the current DNA force fields overstabilise the closed conformations, thus the reopening of the 4WJ into the open cruciform conformation is not observable on a feasible MD timescale (the same issue was seen with pillarplex simulations). The compound was instead observed to bind in the minor groove of the duplex arms, as well as transiently at the duplex termini, with little to no effect on the DNA conformation in both cases (Figure S14). Similar interactions were observed in simulations with a duplex B‐DNA (Figure S15).

### Confirming Binding Through Experiment (PAGE)

To explore these interactions experimentally, both the palladium and platinum versions of the compound were synthesised (Scheme S1). The BIMA ligand and [Pd_2_(BIMA)_4_](NO_3_)_4_ (Pd‐BIMA) were synthesised according to modified literature procedures,^[^ ^25^, ^58^, ^59^ ^]^ while the (new) [Pt_2_(BIMA)_4_](NO_3_)_4_ (Pt‐BIMA) complex was synthesised by mixing the BIMA ligand with Pt(DMSO)_2_Cl_2_ in the presence of AgNO_3_ at 120 °C. Both complexes were obtained as a solid powder after extraction, filtration and drying, and confirmed by ESI mass spectrometry, ^1^H NMR spectroscopy and elemental analysis. In the case of both compounds, and as previously noted by Zhou and Sun for the Pd complex, both enantiomers are discretely present in solution, evidenced by the diastereotopic splitting of the CH_2_^1^H resonance in the NMR spectra (Figures S1, S4). The complexes are soluble at micromolar concentrations – relevant to biophysical studies – in a DMSO water mix (2% DMSO v/v at 20 µM) and at millimolar concentrations, in pure DMSO. UV‐Vis spectroscopy confirms that the complexes are stable in aqueous DMSO over 14 days at room temperature (Figure S8).

**Figure 3:**
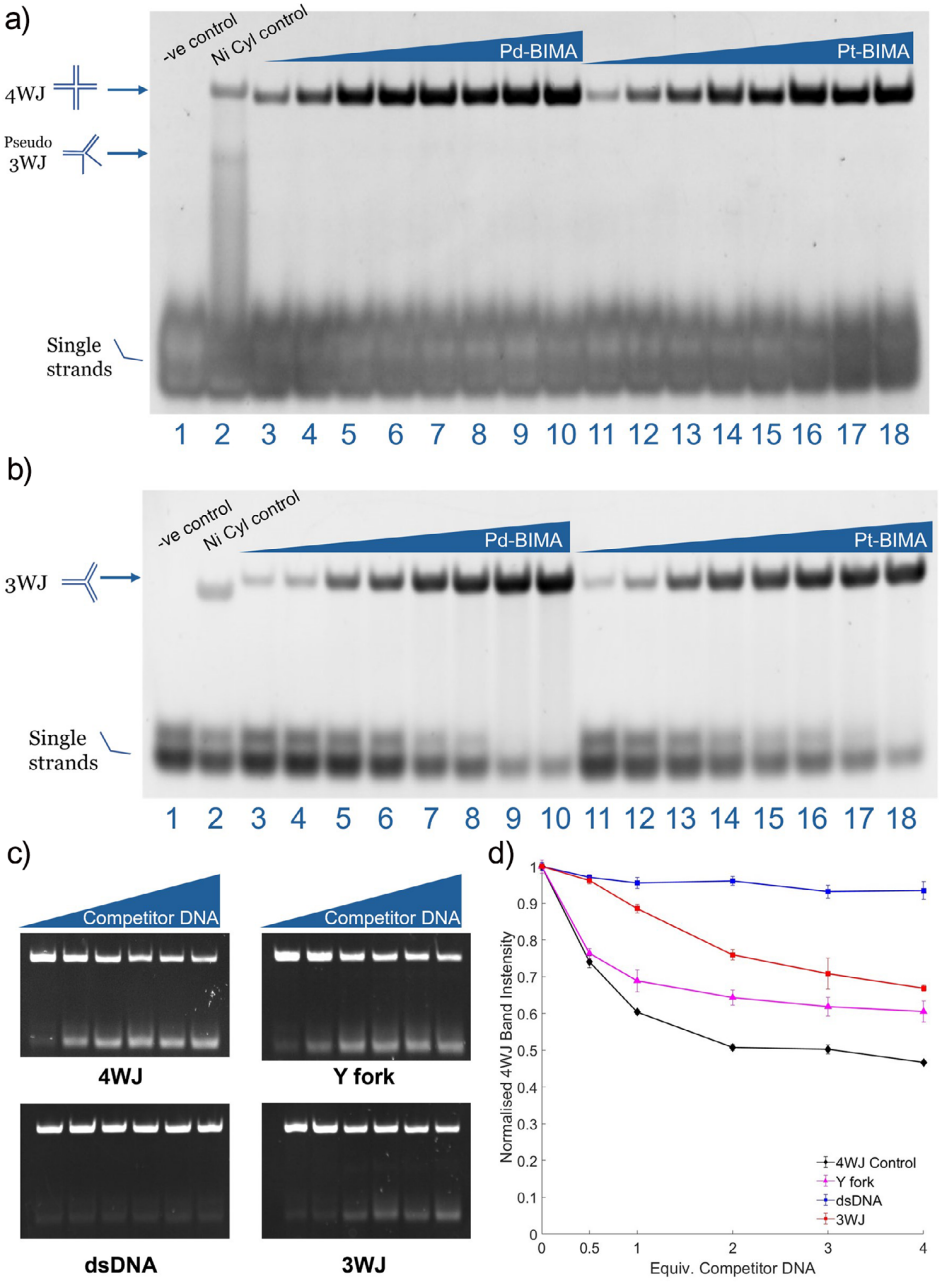
PAGE gels showing binding of Pd‐BIMA and Pt‐BIMA to (a) 4WJ and (b) 3WJ. Gels contains DNA alone (lane 1), DNA + Ni Cylinder (lane 2), DNA + 0.25, 0.5, 1, 1.5, 2, 3, 4, 5 equiv. Pd‐BIMA (lanes 3–10) and DNA + 0.25, 0.5, 1, 1.5, 2, 3, 4, 5 equiv. Pt‐BIMA (lanes 11–18). Gel samples were made up in 1X TB (89 mM Tris base, 89 mM boric acid) and 50Mm NaCl and incubated at 37 °C for 1 h prior to loading. c) Representative PAGE competition gels of 4WJ‐18 versus: 4WJ (control), Y fork, dsDNA and 3WJ. Lanes consist of 4WJ + 1 equivalent Pt‐BIMA with increasing amounts of competitor DNA (0, 0.5, 1, 2, 3, 4 equivalents). Samples were made up in 1X TB and 50 mM NaCl and incubated at 37 °C for 1 h prior to loading. d) Normalised fluorescence intensities of the 4WJ band (represented as % of total fluorescence) in the PAGE competition assays of a fluorescent FAM‐labelled 4WJ‐18 versus 4WJ (control), dsDNA, Y fork and 3WJ DNA at increasing equivalents (0, 0.5, 1, 2, 3, 4). Each data point represents the average of three experiments, normalised to the average fluorescence intensity of the 4WJ band in the absence of competitor.

Polyacrylamide gel electrophoresis (PAGE) experiments were employed to probe the 4WJ binding of the compounds (Figure 3a). Four individual 18 nucleobase DNA strands, designed to form a 4WJ (4WJ‐18) were exposed to increasing equivalents of Pd‐BIMA and Pt‐BIMA. In the absence of any compound, the four strands do not spontaneously assemble into a 4WJ (at room temperature in 50 mM NaCl), instead remaining single stranded. Upon addition of either PdBIMA or PtBIMA, a new, slower‐migrating band appeared, corresponding to a complex‐bound 4WJ species, which became more intense as the loading of compound increased. In a further PAGE experiment with a longer 4WJ comprising 22 bases per strand (4WJ‐22), which does form in the absence of complex, a gel shift in the 4WJ band was observed, indicating binding (Figure S17).

As the stability of 4WJs is influenced by the presence and concentration of cations, further PAGE experiments with both 4WJ‐18 and 4WJ‐22 were conducted at a lower concentration of NaCl of 10 mM (Figure S18) and in the presence of 10 mM MgCl_2_ (Figure S19). Mg^2+^ ions are known to stabilise the *closed* X‐stacked conformation of the 4WJ.^[^ ^60^, ^61^, ^62^ ^]^ The compound was still able to induce formation of the short 4WJ‐18 structure, though the 4WJ band was less bright at the lowest concentration. With the longer 4WJ‐22, there is also evidence of binding, though, rather than a distinct gel shift, the band appears to widen, suggesting a mixture of unbound and bound species, even at the higher concentrations. These suggest that Pt‐BIMA does not bind as well in the presence of Mg^2+^, which may reflect a larger proportion of the 4WJ adopting the closed conformation, though repulsive effects between the divalent cations and the tetracationic complex could also be a contributing factor. PAGE gels with a lower concentration (10 mM) of NaCl also show binding of the compounds in these conditions. Notably, the gel with the 4WJ‐22 was able to resolve bands corresponding to intermediate two‐ and three‐stranded structures bound with complex.

In the light of this possibility of some affinity for other DNA structures, the binding to 3WJs was probed in a similar PAGE experiment (Figure 3b). Like 4WJ‐18, the three individual DNA strands used here do not form a stable 3WJ in the absence of a suitable binding agent. Exposing these three strands to the complex led to the formation of a new band corresponding to a 3WJ + complex species, which became more intense as the loading of compound was increased. Notably, the gel shift of this band was retarded compared to the Ni triple‐helicate cylinder. We have observed the same effect previously with pillarplexes and ascribed this shift to an increase in hydrodynamic radius of the junction, associated with the complex being too large for the 3WJ cavity and so opening up a base pair to enlarge the cavity.^[^ ^24^ ^]^ MD simulations (Figure 6a) support this.

PAGE competition experiments were used to assess the selectivity of the compound for different DNA structures. A 5′ FAM label was added to the S1 strand on 4WJ‐18 allowing the 4WJ to be monitored in the presence of competitor structures. Increasing amounts of competitor DNAs (double‐stranded DNA, Y‐fork, 3WJ) were then titrated against a fixed concentration of 4WJ and 1 equivalent Pt‐BIMA. If the competitor DNA scavenges the compound from the 4WJ, this manifests as a decrease in fluorescence intensity of the 4WJ band on the gel (Figure 3c), which is then measured and normalised as a percentage of the total fluorescence in the sample (Figure 3d). As a control, we use the non‐labelled 4WJ‐18. The assay shows that the 4WJ control leads to the largest decrease in the FAM‐4WJ intensity, indicating that the 4WJ is the preferred DNA structure compared to the other competitors, with the Y‐fork being the next best binder. The 3WJ is a less effective competitor, whilst double‐stranded DNA (dsDNA) showed little to no competition.

### Quantifying the 4WJ Binding

**Figure 4:**
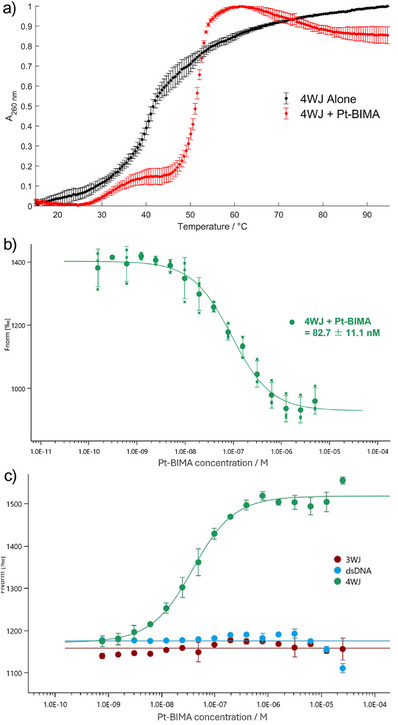
a) UV melting curves of 4WJ with (red) and without (black) 1 equivalent of Pt‐BIMA (10 mM Na cacodylate, 50 mM NaOAc, 0.05% DMSO). Each curve was normalised to its highest and lowest absorbance measurements. Error bars represent standard deviation of three biological replicates. b) MST dose response curve of Pt‐BIMA titrated from 5 µM to 153 pM into 20 nM FAM‐labelled 4WJ‐22. Each data point was measured as the average fluorescence intensity 20 seconds after irradiation of the IR laser and plotted as the average of three repeats. The data was fitted using a K_d_ model and the binding constant taken as the concentration at the halfway point of the curve. c) MST dose response curves corresponding to the competition experiments, in which unlabelled 3WJ (burgundy), dsDNA (blue) and 4WJ (green) were titrated into a mixture of 20 nM FAM‐labelled 4WJ‐22 and 80 nM Pt‐BIMA. The green 4WJ curve was fitted to a displacement model. The straight blue and burgundy lines demonstrate that there is no displacement fit for these curves.

As further confirmation of binding, UV‐Vis melting experiments (monitoring the 260 nm DNA absorbance band with increasing temperature) were performed on the DNA structures with and without complexes (10 mM Na cacodylate, 50 mM NaOAc; Figure 4a). In the absence of compound, this is determined primarily by the hydrogen bonding in the DNA structure. The melting temperature T_m_ of 4WJ‐22 alone is 40.8 ± 0.5 °C. In the presence of 1 equivalent Pt‐BIMA, the curve exhibited a biphasic profile, suggesting a multi‐step melting process (as observed previously for the same 4WJ in the presence of cylinder and pillarplex).^[^ ^24^ ^]^ Using the largest increase of the curve gradient, the T_m_ was calculated to be 52.1 ± 0.1 °C. The ΔT_m_ of 11.6 ± 0.6 °C, both confirms binding of Pt‐BIMA and that the binding stabilises the 4WJ structure. Analogous experiments (Figure S24) also confirm the PAGE results with binding to 3WJ (ΔT_m _= 21.1 ± 0.1 °C) and Y‑fork (ΔT_m _= 5.4 ± 1.1 °C) observed, but no effect on dsDNA melting (ΔT_m _= 0.7 ± 0.9 °C).

To quantify the strength of the interaction between Pt‐BIMA and the 4WJ, microscale thermophoresis (MST)^[^ ^63^ ^]^ was used. Pt‑BIMA was serially diluted from 5 µM to 153 pM and mixed with 20 nM FAM‐labelled 4WJ‐22 (FAM‐4WJ) and a binding curve was obtained by plotting the average intensity of the MST curves 20 seconds after application of the infra‐red laser (Figure 4b, S25), giving a K_d_ of 8.27 ± 1.11 x 10^−8^ M. Pt‐BIMA binds 4WJ more strongly than the organometallic Au pillarplex (K_d_ = 1.91 ± 0.20 x 10^−7^ M) (Figure S26), and MST experiments with dsDNA and Pt‑BIMA did not give a binding curve in the range 50 µM to 1.5 nM Pt‐BIMA, suggesting there is little or no binding affinity for duplex DNA (Figure S27). MST performed with FAM‑4WJ in the presence of non‑labelled dsDNA (20 nM, 1 equivalent) gave K_app_ = 8.92 ± 0.63 × 10^−8^ M (Figure S28), consistent with the PAGE competition assay in showing that dsDNA is not a competitor for Pt‐BIMA. MST was also used to calculate K_d_ = 1.90 ± 2.27 × 10^‑6^ M for the binding of Pt‐BIMA with the 3WJ (Figure S29), and K_app_ = 2.41 ± 0.64 × 10^−7^ M for Pt‑BIMA to the 4WJ in the presence of (non‐labelled) 3WJ (Figure S30).

Competition was then assessed further by titrating competitor DNA (3WJ, dsDNA and 4WJ control) into a fixed concentration of FAM‐4WJ and Pt‐BIMA, where changes in the MST traces correspond to release of Pt‐BIMA from the 4WJ, in response to the competitor (Figures 4c, S31). The validity of this approach was first confirmed by titrating in unlabelled 4WJ and observing an IC_50_ of 2.88 ± 0.42 x 10^−8^ M. In contrast, no binding curve was observed with dsDNA or 3WJ (IC_50_ > 25 µM), confirming the PAGE competition results that the 4WJ is the preferred. These MST results are consistent with the PAGE experiments and confirm that Pt‐BIMA binds most strongly to 4WJs.

### Location and Nature of Binding

**Figure 5:**
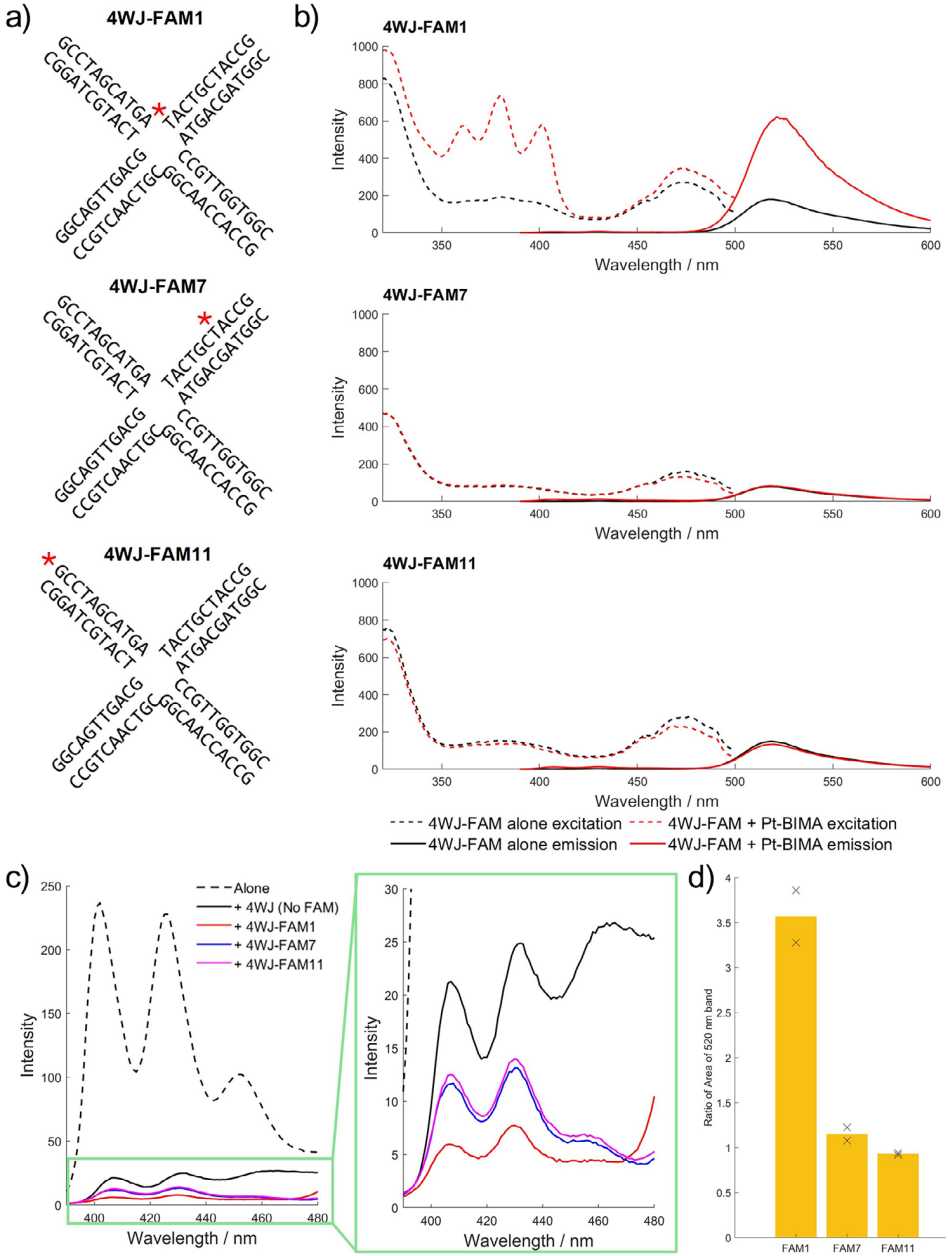
a) Schematic drawing of the fluorescently labelled 4WJ structures (4WJ‐FAM). The red star denotes the position of the fluorophore. b) Fluorescence excitation and emission spectra for (top to bottom) 4WJ‐FAM1, 4WJ‐FAM7 and 4WJ‐FAM11 in the absence (black) and presence (red) of 1 equivalent Pt‐BIMA (all 5 µM DNA, 10 mM HEPES, 50 mM NaOAc). Excitation spectra were recorded by monitoring the emission at 520 nm with a 475 nm cutoff filter. Emission spectra were recorded by exciting at 375 nm. Spectra for control samples containing Pt‐BIMA alone and with unlabelled 4WJ, and corresponding absorbance spectra for all samples can be seen in the supporting information (Figures S33, S34). c) Zoom in and overlay of the emission curves of Pt‐BIMA in the region 390–480 nm, where Pt‐BIMA emits. The portion of the graph in the green box shows a further zoom in on the quenched anthracene bands. d) Bar chart showing the ratio of the 520 nm FAM emission band in the presence of Pt‐BIMA to the same band in the absence of Pt‐BIMA, measured as the area under the curve. The bars represent the average of two samples, and the black crosses represent the individual datasets (for FAM11, they overlap).

Pt‐BIMA exhibits strong emission in the region of 400–500 nm, with characteristic anthracene emission bands at 402, 426 and 452 nm (Figures 5c and S32). On addition of 4WJ DNA this emission is reduced. Quenching of anthracene emission (and changes in the relative intensities of the three bands) on DNA‐binding have been reported for simple anthracene intercalators, and so this effect is consistent with the complex inserting into the 4WJ.^[^ ^64^, ^65^ ^]^ A time‐resolved emission profile (using a 375 nm excitation laser) of the Pt‐BIMA complex alone exhibited biexponential behaviour, with one decay profile attributed to typical organic ligand fluorescence with a lifetime *τ* = 3.41(2) ns. A shorter lifetime component with a lifetime on the order of roughly 100 ps is tentatively attributed to an ICT band. When 4WJ DNA is present, the long lifetime component appears entirely quenched (Figure S35A), consistent with strong interaction between Pt‐BIMA and the DNA, and with the drop in the complex emission intensity.

The Pt‐BIMA emissions have good spectral overlap with the excitation band of the FAM (fluorescein) dye (J = approx. 5 x 10^14^ nm^4^ M^−1^ cm^−1^), offering the potential for FRET. Therefore, to probe the location of Pt‐BIMA binding, three new oligos were designed, incorporating a FAM tag at different positions of the 4WJ‐22 S1 strand: attached to the thymine at the junction branchpoint (4WJ‑FAM1), to a thymine 7 bases away from the branchpoint (4WJ‐FAM7), and at the 5′ end (4WJ‐FAM11) (Figure 5a). Each oligo was mixed with the complementary strands in buffer (10 mM HEPES, 50 mM NaOAc) to form the 4WJ, either in the absence or presence of 1 equivalent Pt‐BIMA, and the fluorescence excitation and emission spectra recorded.

When the FAM label is positioned closest to the central 4WJ branchpoint (4WJ‐FAM1), on addition of Pt‐BIMA and irradiation at 375 nm, the Pt‐BIMA emission reduces, while the FAM emission intensifies approximately 3.5‐fold (calculated as the area under the 520 nm band). Crucially, the excitation spectrum of the FAM emission now shows the Pt‐BIMA anthracene absorption bands, revealing a clear FRET interaction (Figure 5b–d). By contrast, there is little or no increase in the FAM fluorescence, nor anthracene contribution to the excitation spectrum, when the FAM label is positioned either in the middle of the duplex arm (4WJ‑FAM7), or at the end of the strand (4WJ‐FAM11). This is clear evidence for the Pt‐BIMA compound being bound at the heart of the 4WJ.

The quenching observations with unlabelled 4WJ, taken together with the red‐shift in complex absorption on binding to the 4WJ (Figure S34), demonstrate that the Pt‐BIMA ligands are stacked with the DNA bases. Given the FRET confirms that Pt‑BIMA is located at the junction branchpoint, the experimental observations that the anthracene ligands are stacked on the bases suggests that the complex has entered inside the junction cavity. To accommodate the dimensions of the drug, this in turn implies that the junction cavity is in its open conformation. This is reinforced by the MD simulations that indicate that Pt‐BIMA does not associate with the junction branchpoint when it is closed, only when it is open (Figures 2a, S14). Single‐molecule FRET experiments with Atto550 and Atto647N‐labelled 4WJs (see SI) were hampered by fluorophore quenching at high Pt‐BIMA loading.

### Affinity for Bulged 3WJs

**Figure 6:**
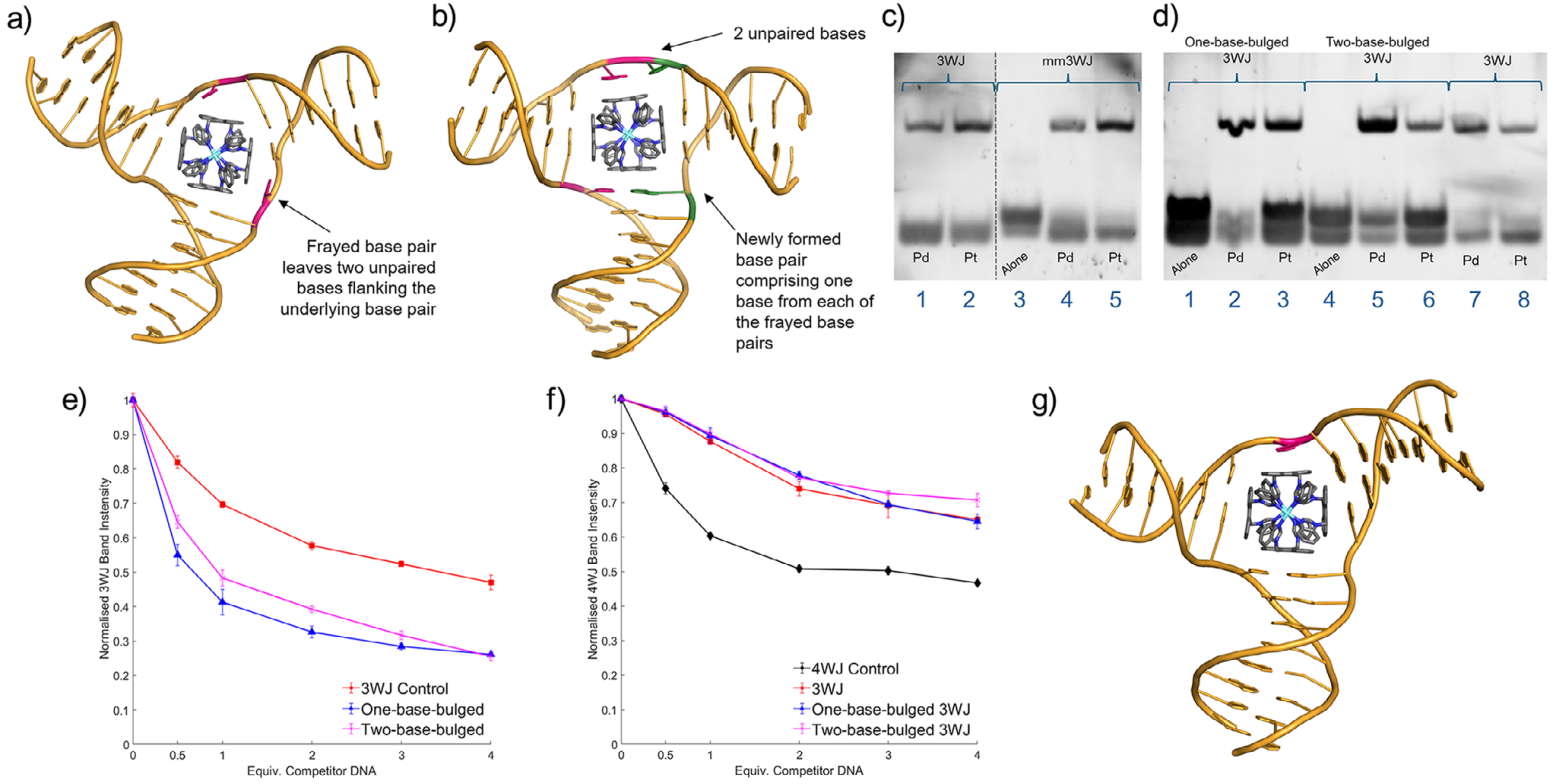
a) MD snapshot of the M enantiomer of Pt‐BIMA bound inside a frayed 3WJ (frayed base pairs shown in pink). Hydrogens are omitted for clarity. b) MD snapshot of the M enantiomer bound to a T‐shaped two‐base‐bulged 3WJ after rearrangement caused by fraying of two base pairs (shown in pink and green). Hydrogens are omitted for clarity. c) PAGE gel showing binding of Pd‐BIMA and Pt‐BIMA to a bulged 3WJ. Gel contains 3WJ (lanes 1–2), mismatched mm3WJ (lanes 3–5). The dotted line represents a splice point, as three lanes of this gel are not included for clarity (full raw image Figure S36). d) PAGE gel showing binding of Pd‐BIMA and Pt‐BIMA to bulged 3WJs. Gel contains one‐base‐bulged 3WJ (lanes 1–3), two‐base‐bulged 3WJ (lanes 4–6) and 3WJ (lanes 7–8; aliquots of same samples used in lanes 1 and 2 of Figure 6c). The gels in parts 6c and 6d were run in parallel under identical conditions (details in Figure S36). e) Plot of normalised intensity of the 3WJ band upon increasing additions of competitor 3WJ S1 (i.e., zero‐base‐bulged), one‐base‐bulged 3WJ S1 and two‐base‐bulged 3WJ S1. Each data point is plotted as the average of at least three independent samples with associated error bars. f) Normalised fluorescence intensities of the 4WJ band (represented as % of total fluorescence) in PAGE competition assays of a fluorescent FAM‐labelled 4WJ versus 4WJ (control), 3WJ, one‐base‐bulged 3WJ and two‐base‐bulged 3WJ DNA at increasing equivalents (0, 0.5, 1, 2, 3, 4). Each data point represents the average of three experiments, normalised to the average fluorescence intensity of the 4WJ band in the absence of competitor. g) MD snapshot of the M enantiomer of Pt‐BIMA bound inside a one‐base‐bulged 3WJ (adapted from PDB 1F44). The unpaired base is highlighted in pink. Hydrogens are omitted for clarity.

Despite the compound's preference for 4WJs over 3WJs, we were interested to use MD to provide insight into how the compound might interact with 3WJs, given the poor size and shape match. MD simulations starting with PtBIMA close to, but outside, the 3WJ cavity, all (3 per enantiomer at 2 µs each) captured the entry of the compound into the cavity. As expected from the PAGE gel shift, a branchpoint base pair frayed in all cases (leaving one unpaired base on two of the three strands), in order to accommodate Pt‐BIMA within the cavity (Figure 6a). In this conformation, the compound remained inside the cavity, though there is much movement of the compound as it attempts to align its anthracene units with the base pairs but is unable to adopt a unique binding position because there are not four base pairs available to the four anthracenes. Occasionally observed was a partial melting of a duplex arm and folding over of one of the strands, allowing the terminal base to interact with the compound (Figure S16).

In one simulation with the M enantiomer, fraying of two branchpoint base pairs led to the rearrangement of the junction into a T‐shaped two‐base‐bulged 3WJ, wherein two unpaired nucleotides are present on one strand (Figure 6b). This conformation mimics the tetragonal shape of the 4WJ cavity very well, allowing three base pairs to *π* stack with 3 of the anthracene units on the compound, analogous to the 4WJ binding, with two unpaired bases stacking dynamically on the 4th anthracene unit. Due to sequence differences, the 3WJ used in the experimental studies should not be able to rearrange in the same way, as there is no valid Watson‐Crick base pairing available after double base pair fraying to form the new base pair opposite the unpaired bases. However, this did stimulate us to explore whether Pt‐BIMA might bind other different 3WJ structures.

Three new sequences were considered, derived from the original 3WJ sequence (Figure 2b): a mismatched 3WJ (analogous to a frayed 3WJ), a one‐base‐bulged 3WJ and a two‐base‐bulged 3WJ. PAGE confirmed that both Pd‐BIMA and Pt‐BIMA bind to all of these structures (Figure 6c,d). The gel shift of the complexes with mismatched 3WJ is found at the same position as that with the “perfect” 3WJ, which is consistent with the complexes opening the cavity and causing base pair fraying upon “perfect” 3WJ binding.

A strand displacement competition assay was conducted to investigate and compare the affinity of the compound for bulged 3WJ structures utilising a FAM‐labelled 3WJ S1 strand. Upon titration of nonlabelled S1 strands corresponding to the one‐base and two‐base‐bulged 3WJs, the labelled strand is displaced and the change in the fluorescence of the 3WJ PAGE band can subsequently be measured (Figure S23). Interestingly, Pt‐BIMA showed a preference for both the one‐base‐ and two‐base‐bulged 3WJs, displacing 59% and 52%, respectively (compared to 30% in the control) at 1 equivalent (Figure 6e). At low equivalents, the one‐base‐bulged 3WJ appears to be the slightly more favourable substrate for the compound, though at higher equivalents they compete near equally. Importantly, a PAGE competition assay of the bulged 3WJs against the 4WJ showed that they are not good competitors and the 4WJ is still preferred (Figure 6f).

To explore why binding to the one‐base‐bulged 3WJ might be favoured we conducted further simulations. A one‐base‐bulged 3WJ structure file was generated from a snapshot of the two‐base‐bulged 3WJ that emerged in the earlier simulation by removing Pt‐BIMA, deleting the unpaired thymine base and energy minimising the structure to rejoin the DNA strand. Pt‐BIMA was then reinserted into the cavity and the MD simulations started from here (3 per enantiomer, 1.2 µs each). The simulations showed that Pt‐BIMA is well accommodated in the cavity, which does not need to expand by base pair fraying (Figure 6g). As expected, the M enantiomer is better able to align its anthracene units with the three base pairs than the P enantiomer and hence finds a less dynamic binding mode, though both remain inside the junction for the duration of the simulation in all cases. The unpaired base is then able to bind (stably with the M enantiomer, dynamically with the P enantiomer) with the remaining anthracene unit. Notably, the cavity of the one‐base‐bulged 3WJ is more trapezoidal than the preferred square shape of the 4WJ – which the two‐base‐bulged 3WJ better mimics – however the presence of only one unpaired base reduces the cavity size and ultimately provides a more snug fit for PtBIMA; in other words, the one‑base‑bulged 3WJ provides a more optimal size match, at the expense of shape matching, whilst the two‐base‐bulged 3WJ provides the reverse. The subtle effects of both cavity size and cavity shape rationalise why there is little difference in the preference of PtBIMA for one‐base‐ and two‐base‐bulged 3WJs and additionally further highlight that the 4WJ is an excellent binder for these compounds, possessing both of these qualities.

## Conclusion

To conclude, we have shown that a quadruple‐stranded metallo‐cage with an anthracene presenting surface is the right shape and size to bind to the cavity of 4WJs, and a better fit than previously studied pillarplexes. This is a new frontier for these popular square‐planar metallo‐cages in which the activity is not about the interior of the cage but now about its external surface. It opens future possibilities to use both the inside and the outside in tandem. The work also expands the types of available DNA junction binding agents (adding quadruple‐helicates as DNA‐binders) and confirms fundamental design principles for supramolecular compounds to bind in the heart of nucleic acid structures and lock those structures in their open form.

Importantly, the work demonstrates that the approach of positioning external aryl surfaces for nucleic acid junction‐binding is effective, and that available structural databases can be used to identify suitable binders for DNA junctions. Once identified, a potential binder can be screened in silico for its fit to a nucleic acid junction target using MD simulations. Together, this has potential to significantly accelerate discovery in this area.

While 4WJs are the preferred binding partner, Pt‐BIMA can also bind to 3WJs, though more weakly. The stability of this binding can be influenced by the incorporation of unpaired bases into the cavity. This shows how relatively subtle effects and careful design should enable agents with a preference for a precise DNA micro‐structure (such as T‐shape or Y‐shape 3WJs). It also illustrates the importance of the size‐shape match between junction substrates and binders, and reinforces why the BIMA complexes have a selectivity for 4WJs.

For many biological applications, it is a positive feature for agents to target several different related junction structures – DNA repair processes invoke Y‐forks, 3WJ and 4WJ all of which are targets,^[^ ^8^, ^9^, ^10^, ^11^ ^]^ while viral RNA genomes present a variety of junction and junction‐like structures in their regulatory regions (UTRs) which are crucial to viral replication and action.^[^ ^35^ ^]^ Nevertheless, as this recognition approach advances to higher order nucleic acid structures, achieving exclusive binding may be a challenge if the agent is simply able to rearrange lower order structures to create larger cavities. Coupling to other recognition events offers potential to meet this challenge, and we are exploring this together with the wider potential of the BIMA complexes in biology.

## Supporting Information

Supporting information is available comprising experimental synthesis and characterisation details, experimental biophysical details and additional gels, UV and fluorescence spectra, MST traces, TCSPC plots and further details of and images from the MD simulations. Original data and videos of the MD simulations are available at the University of Birmingham data repository UBIRA (https://doi.org/10.25500/edata.bham.00001248). The authors have cited additional references within the Supporting Information.^[^ ^66^, ^67^, ^68^, ^69^, ^70^, ^71^, ^72^, ^73^, ^74^, ^75^, ^76^, ^77^, ^78^, ^79^, ^80^, ^81^ ^]^

## Conflict of Interests

The authors declare no conflict of interest.

## Supporting information



Supporting Information

Supporting Information

Supporting Information

Supporting Information

## Data Availability

The data that support the findings of this study are openly available in the University of Birmingham data repository UBIRA at doi.org/10.25500/edata.bham.00001248.
