# Supplementary material for: Supramolecular Recognition of a DNA Four‐Way Junction by an M2L4 Metallo‐Cage, Inspired by a Simulation‐Guided Design Approach
Source: Angew Chem Int Ed Engl. 2025 May 5;64(26):e202504866. doi: 10.1002/anie.202504866 (PMC12184311; doi:10.1002/anie.202504866)
Supplement: Supplementary file 1 — Supporting Information [file ANIE-64-e202504866-s002.pdf]

Angewandte Chemie
Eine Zeitschrift der Gesellschaft Deutscher Chemiker

Supporting Information

Supramolecular Recognition of a DNA Four-Way Junction by an M_2L_4 Metallo-Cage, Inspired by a Simulation-Guided Design Approach

*Samuel J. Dettmer, Hugo D. Williams, Richard Napier, Joseph M. Beames, Steffan Walker-Griffiths, Timothy D. Craggs, Michael J. Hannon**

In addition to the Supplementary Information herein we have made available all raw NMR, mass spectrometry, IR, gel electrophoresis, UV-Vis, MST and simulation data, as well as videos for the simulations, online at: 10.25500/edata.bham.00001248

SUPPLEMENTARY INFORMATION

TABLE OF CONTENTS

Contributions 3

Experimental Methods 4

- **Scheme S1.** Reaction scheme for the synthesis of the BIMA complexes 4
- Synthesis of 9,10-bis(bromomethyl)anthracene 4
- Synthesis of BIMA ligand 4
- Synthesis of Pd-BIMA 5
- **Figure S1.** ^{1}H NMR spectrum for Pd-BIMA 6
- **Figure S2.** COSY spectrum for Pd-BIMA 6
- **Figure S3.** nESI-MS spectrum for Pd-BIMA 7
- Synthesis of $Pt(DMSO)_2Cl_2$ 8
- Synthesis of Pt-BIMA 8
- **Figure S4.** ^{1}H NMR spectrum for Pt-BIMA 9
- **Figure S5.** COSY spectrum for Pt-BIMA 9
- **Figure S6.** nESI-MS spectrum for Pt-BIMA 10
- DNA Sequences 11
- Polayacrylamide Gel Electrophoresis (PAGE) 11
- PAGE Competition Experiments 12
- Fluorescence Spectroscopy 12
- UV-Visible Spectroscopy 12
- UV Melting 13
- Microscale Thermophoresis (MST) 13
- Time-Correlated Single Photon Counting (TCSPC) 13
- Single Molecule FRET 13

Molecular Dynamics Simulations 13

- Parameterisation of Pd-BIMA and Pt-BIMA 13
- Parameterisation of DNA 14
- Simulations 14

Supplementary Data 15

- **Figure S7.** UV-VIS spectra of Pd- and Pt-BIMA 15
- **Figure S8.** Stability of Pd- and Pt-BIMA in solution 15
- **Figure S9.** Comparison of Pd-BIMA and iron cylinder 16
- **Figure S10.** Comparison of Pd-BIMA and Au pillarplex 16
- **Figure S11.** Comparison of Pd- and Pt-BIMA DFT structures 16
- **Figure S12.** Zoom in of MD snapshot of Pt-BIMA in 4WJ cavity 17
- **Figure S13.** RMSD plots for representative 4WJ simulations 17
- **Figure S14.** MD snapshot of Pt-BIMA with closed X-stacked 4WJ 18
- **Figure S15.** MD snapshot of Pt-BIMA with B-DNA 18
- **Figure S16.** MD snapshot of Pt-BIMA with 3WJ exhibiting partial melting 18
- **Figure S17.** PAGE gel of Pd- and Pt-BIMA with 4WJ-22 in 50 mM NaCl 19
- **Figure S18.** PAGE gel of Pd- and Pt-BIMA with 4WJ-22 in 10 mM NaCl 19
- **Figure S19.** PAGE gels of Pt-BIMA with 4WJ in Na and Mg conditions 20

Figure S20. PAGE gel of Pt-BIMA with all strand combinations of 4WJ-18 21
Figure S21. PAGE gel of Pt-BIMA with all strand combinations of 4WJ-22 21
Figure S22. PAGE gels of the free BIMA ligand and Ru(Phen)$_3$Cl$_2$ with 4WJs 22
Figure S23. Representative gels from each PAGE displacement or competition assay 23
Figure S24. UV melting curves for3WJ, Y fork and dsDNA 23
Figure S25. MST data for 4WJ + Pt-BIMA 24
Figure S26. MST data for 4WJ + Au pillarplex 25
Figure S27. MST data for dsDNA + Pt-BIMA 26
Figure S28. MST data for 4WJ + Pt-BIMA in the presence of dsDNA 27
Figure S29. MST data for 3WJ + Pt-BIMA 28
Figure S30. MST data for 4WJ + Pt-BIMA in the presence of 3WJ 29
Figure S31. MST data for the competition titrations 30
Figure S32. Fluorescence spectra for Pd- and Pt-BIMA 31
Figure S33. Fluorescence spectra for controls for experiments shown in Fig 5B 31
Figure S34. Absorbance spectra for experiments shown in Figs 5B and S31 32
Figure S35. TCSPC plots 32
Figure S36. Raw gel image for figures 6C and 6D 33
Further discussion on the TCSPC data 33

References 34

CONTRIBUTIONS

MJH, HDW and SJD conceived the project which MJH supervised. SJD synthesised the compounds, with assistance from HDW, and undertook DFT, MD simulations and biophysical studies (gels, UV and fluorescence spectroscopy, MST, single molecule experiments). RN supervised MST experiments and assisted with their analysis, and TDC supervised single molecule experiments, while SWG and JMB undertook and analysed fluorescence lifetime studies. SJD and MJH analysed the data and wrote the manuscript which all authors discussed and commented on.

EXPERIMENTAL METHODS

All solvents, NMR solvents, chemical reagents and buffer components, and $[Ru(phen)_3]Cl_2$, were purchased from Fischer Scientific, VWR chemicals or Sigma Aldrich and used without further purification. Nickel cylinder $[Ni_2L_3]Cl_4$ was prepared as described previously.[1] Au pillarplex was prepared as previously described and kindly provided by Alex Pöthig and colleagues.[2] Electrospray ionisation (ESI) mass spectrometry characterisation was carried out on a Waters SYNAPT-G2-S in positive ion mode. ^1H NMR studies were carried out on AVIII 300 (300 MHz), AVANCE NEO400 and AVIII 400 (400MHz) Bruker spectrometers. Elemental analysis was performed on a CE Instruments EA1110 elemental analyzer. IR spectra were recorded on a PerkinElmer Spectrum Two FT-IR spectrometer. Nuclease free water was used in all biophysical experiments.

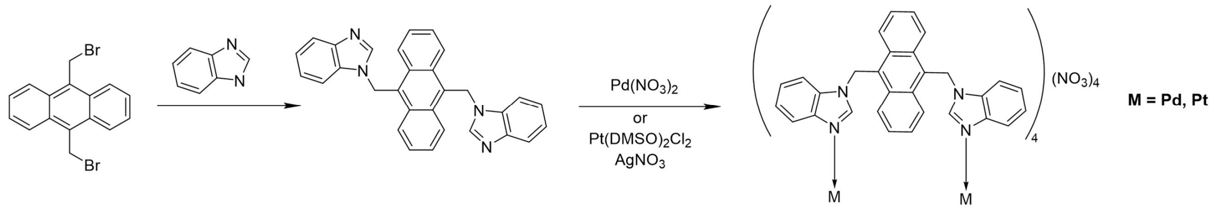

Scheme S1. Reaction scheme for the synthesis of Pd-BIMA and Pt-BIMA.

Synthesis of 9,10-bis(bromomethyl)anthracene

9,10-bis(bromomethyl)anthracene was synthesised as previously reported.[3] Anthracene (5 g, 28.05 mmol, 1 equiv.), paraformaldehyde (1.654 g, 56.11 mmol, 2 equiv.) and cetyltrimethylammonium bromide (CTAB) (0.095 g) were stirred in acetic acid (7 mL) at room temperature for 1 hour. Over 15 minutes, aqueous HBr (48 w%, 7 mL, 3.8 equiv.) was added dropwise to the reaction mixture. The reaction mixture was then heated at 80 °C for 16 hours. The reaction mixture was cooled and the solid collected by filtration, washed with water and dried in a vacuum desiccator. The yellow solid was then recrystallised from toluene. Yield 3.575 g (35%).

^1H NMR (300 MHz, dichloromethane-d_2) δ 8.40 (dd, J = 6.9, 3.3 Hz, 4H), 7.70 (dd, J = 6.9, 3.2 Hz, 4H), 5.56 ppm (s, 4H).

Synthesis of 9,10-bis(benzimidazolylmethyl)anthracene (BIMA)

BIMA was synthesised according to an adapted literature procedure.[4] Benzimidazole (194.7 mg, 1.65 mmol, 1 equiv.) and NaOH (66 mg, 1.65 mmol, 1 equiv.) were dissolved in DMF (8 mL) and stirred for 1 hour at 60 °C. 9,10-bis(bromomethyl)anthracene (300 mg, 0.82 mol, 0.5 equiv.) was then added and the reaction heated at 80 °C for 18 hours. The reaction mixture was then cooled and poured into aqueous NaOH (2 M, 20 mL). The yellow solid was collected by filtration, washed with water, acetone and diethyl ether and dried in a vacuum desiccator, leaving a pale-yellow solid. Yield 224 mg (62%).

^1H NMR (400 MHz, DMSO-d_6) δ 8.58 – 8.51 (m, 4H), 7.78 (s, 2H), 7.67 – 7.55 (m, 8H), 7.27 – 7.14 (m, 4H), 6.53 ppm (s, 4H).

ESI: m/z $[BIMA + H]^+$ 439.1891 (calc 439.1923); ($C_{23}H_{17}N_2^+$) 321.1438 (calc 321.1392).

Synthesis of Pd-BIMA

Pd-BIMA was synthesised as previously reported. [5] $Pd(NO_3)_2.2H_2O$ (14 mg, 0.05 mmol, 1 equiv.) was dissolved in 1 mL DMSO. To this, BIMA (44 mg, 0.10 mmol, 2 equiv.) was added and the mixture stirred and heated to 70 °C for 2 hours. The reaction mixture was then cooled and transferred into a 50 mL centrifuge tube. The tube was topped up to 50 mL with diethyl ether, shaken to precipitate the solid, and then centrifuged. The supernatant was then removed, and the tube topped up again with diethyl ether, repeating the cycle of washing, centrifugation and supernatant removal until the solid became a fine powder. The solid was then washed once with DCM and once with water in the same way. The solid was then collected by filtration and dried over P_2O_5 in a vacuum desiccator overnight. Yield 37 mg (66%).

1H NMR (400 MHz, DMSO-*d6*) δ 8.70 (d, J = 9.1 Hz, 8H), 8.24 (d, J = 8.2 Hz, 8H), 7.98 (br dd, 8H), 7.78 (m, 8H), 7.46 (d, J = 4.1 Hz, 16H), 6.53 – 6.38 (m, 16H), 6.31 (d, J = 15 Hz, 8H), 6.06 (s, 8H), 5.48 ppm (dd, J = 9.1, 6.4 Hz, 8H).

ESI: *m/z* $[Pd_2L_4](NO_3)^{3+}$ 676.1838 (calc 676.1792); $[Pd_2L_4](NO_3)_2^{2+}$ 1045.2583 (calc 1045.2627).

Elemental analysis: (%) Calculated for $Pd_2C_{120}H_{88}N_{20}O_{12}\cdot 5H_2O$: C 62.53, H 4.29, N 12.15; Found C 62.75, H 4.11, N 12.10.

IR: (cm^{-1}) 3444 (br), 3111 (w), 1614 (w), 1521 (m), 1465 (m), 1343 (s), 1229 (s), 1195 (m), 1011 (w), 934 (w), 813 (w), 745 (s), 694 (s), 691 (m), 657 (m), 591 (m), 542 (m), 476 (m).

UV-VIS (H_2O, 2% DMSO): 399 (ε = 27,600), 378 (ε = 25,400), 358 (ε = 15,900), 341 (ε =7,190), 324 nm (ε = 2,990 $M^{-1}cm^{-1}$).

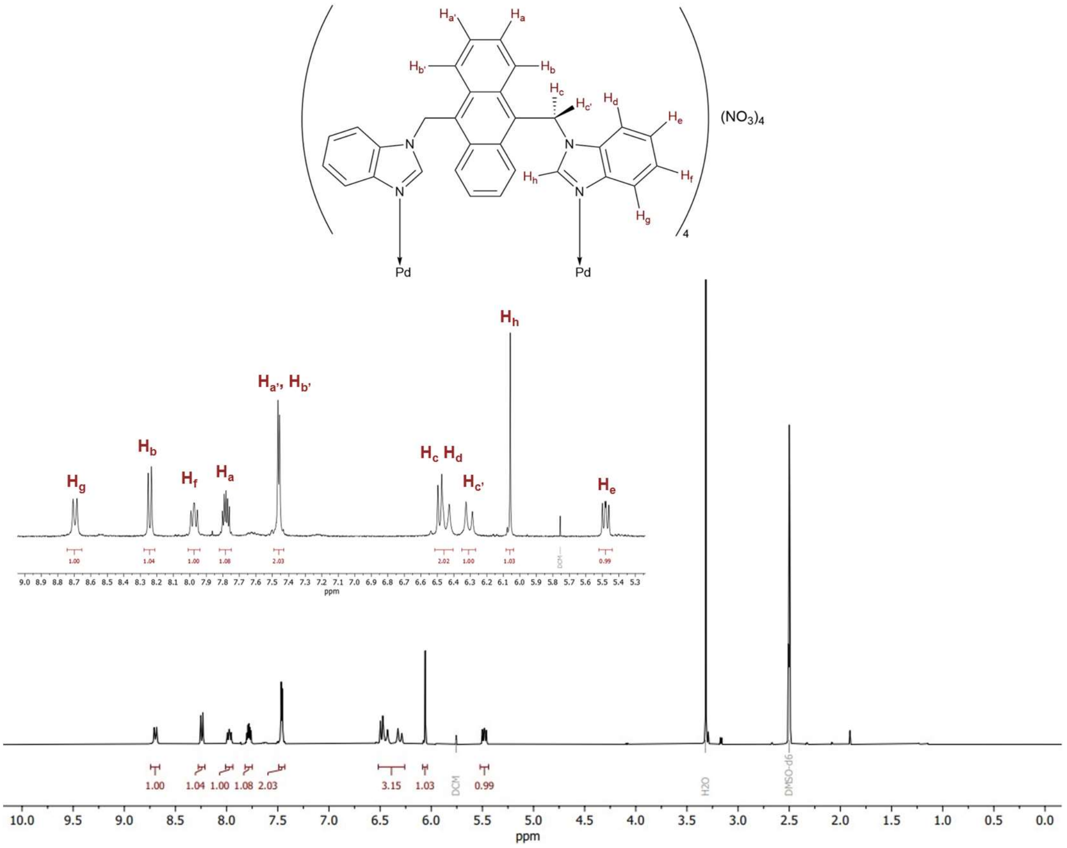

Figure S1. 1H NMR spectrum of Pd-BIMA in DMSO-*d*6. A zoom in of the region 5.2 – 9.0 ppm is included. Assignments used COSY experiments which confirm the ring connectivity but there is potential ambiguity in the direction of the ring system assignments (e.g. g/d and e/f).

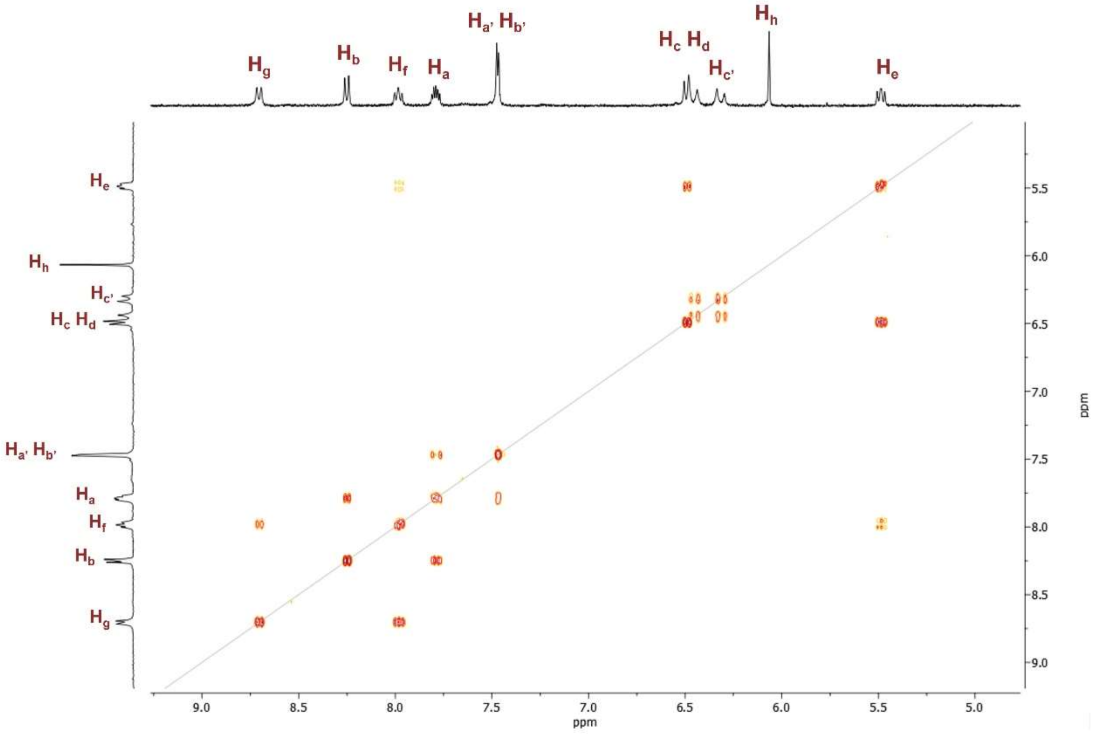

Figure S2. COSY NMR spectrum of Pd-BIMA in DMSO-*d*6 used to assign the ^{1}H peaks (400 MHz).

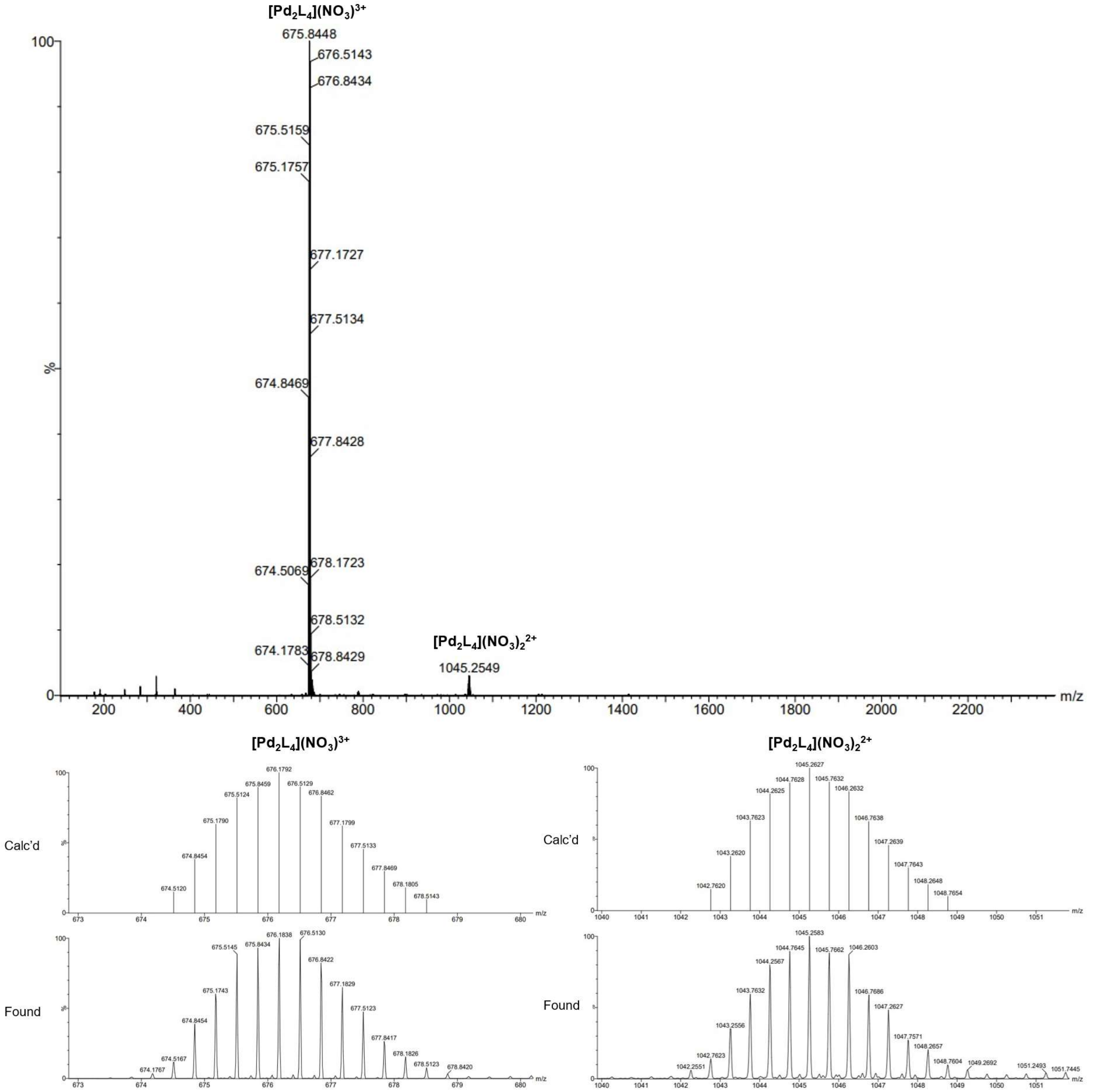

Figure S3. nESI-MS spectrum of Pd-BIMA in acetone. Below are zoom ins of relevant peaks and the corresponding simulated peaks.

Synthesis of $Pt(DMSO)_2Cl_2$

The platinum precursor was synthesised as previously reported.[6] K_2PtCl_4 (925 mg, 2.23 mmol, 1 equiv.) was dissolved in minimal H_2O (5 mL). To this, DMSO (633 µL, 8.91 mmol, 4 equiv.) was then added and the mixture stirred at room temperature for 1 hour before being allowed to stand overnight. The resultant off-white precipitate was collected by filtration, washed with water, ethanol and diethyl ether, and then dried in a vacuum desiccator. Yield 866 mg (92%)

^{1}H NMR (400 MHz, DMSO-d_6) δ 2.54 (s, 6H).

Synthesis of Pt-BIMA

$Pt(DMSO)_2Cl_2$ (19 mg, 0.046 mmol, 1 equiv.) was dissolved in 1 mL DMSO. To this, BIMA (40 mg, 0.091 mmol, 2 equiv.) and $AgNO_3$ (15 mg, 0.09 mmol, 1.98 equiv.) was added and the mixture stirred and heated to 120 °C for 24 hours. The reaction mixture was then cooled and transferred into a 50 mL centrifuge tube. The tube was topped up with diethyl ether, shaken to precipitate the solid, and then centrifuged. The supernatant was then removed and the tube topped up again with diethyl ether, repeating the cycle of washing, centrifugation and supernatant removal until the solid became a fine powder. The solid was then washed 2 times with DCM (50 mL) in the same way. The solid was collected by filtration, washed further with 3-5 mL dichloromethane and water and dried over P_2O_5 in a vacuum desiccator overnight. Yield 11mg (51%)

^{1}H NMR (400 MHz, DMSO-*d6*) δ 8.74 (d, *J* = 9.1 Hz, 7H), 8.26 (d, *J* = 8.2 Hz, 8H), 8.00 (br dd, 8H), 7.78 (br t, 8H), 7.55 – 7.44 (m, 16H), 6.49 (dd, *J* = 15, 8.7 Hz, 16H), 6.46 (d, J~9Hz, 8H), 6.34 (d, *J* = 15 Hz, 8H), 6.14 (s, 8H), 5.49 (dd, *J* = 9.1, 6.4 Hz, 8H).

ESI: *m/z* $[Pt_2L_4](NO_3)^{3+}$ 735.2159 (calc 735.2192); $[Pt_2L_4](NO_3)_2{}^{2+}$ 1133.8177 (calc 1133.8226); $[[Pt_2L_4](NO_3) - H]^{2+}$ 1102.3275 (calc 1102.3248).

Elemental analysis: (%) Calculated for $Pt_2C_{120}H_{88}N_{20}O_{12}\cdot 4CH_2Cl_2$: C 54.51, H 3.54, N 10.25; Found C 54.33, H 3.67, N 9.97.

IR: (cm^{-1}) 3434 (br), 3107 (w), 1615 (w), 1524 (m), 1465 (m), 1353 (m), 1299 (m), 1230 (m), 1194 (m), 1041 (w), 1037 (m), 939 (m), 815 (m), 745 (s), 695 (m), 657 (m), 600 (m), 543 (m), 479 (m).

UV-VIS (H_2O, 1% DMSO): 399 (ε = 32,000), 378 (ε = 29,100), 358 (ε = 18,500), 341 (ε = 8,750), 324 nm (ε = 4,050 M^{-1}cm^{-1}).

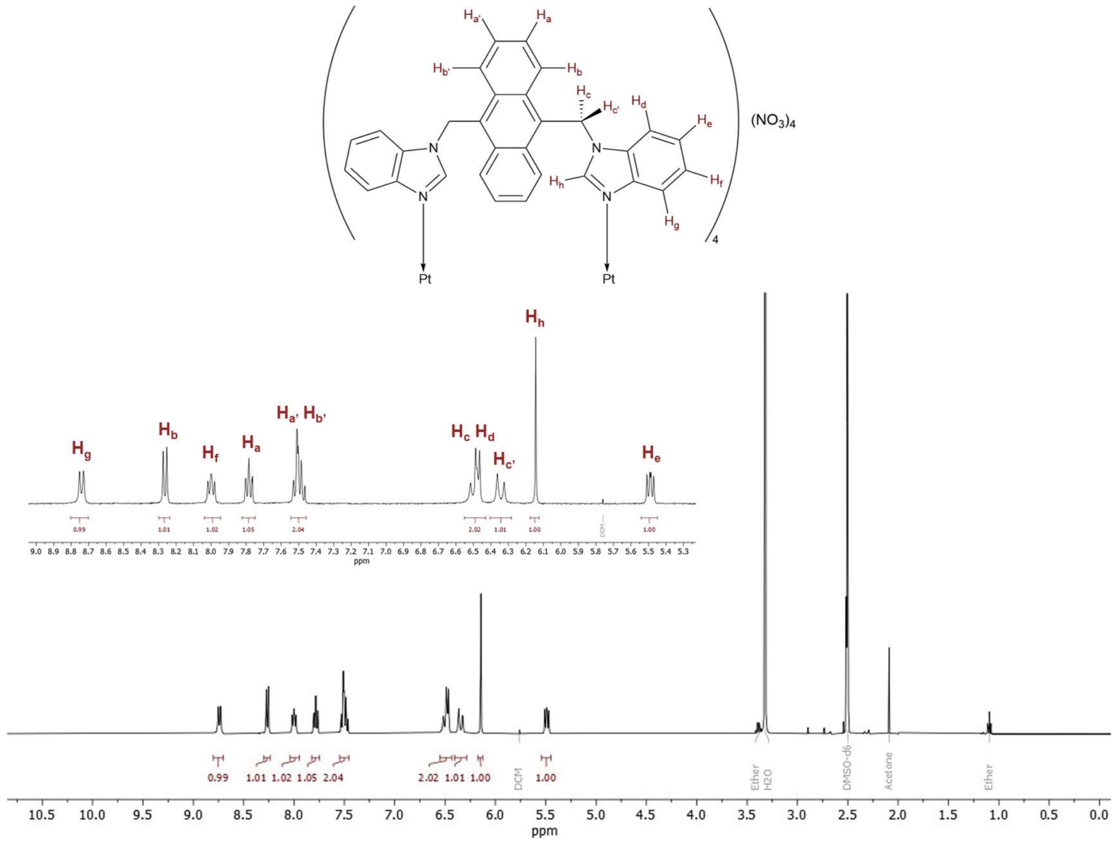

Figure S4. 1H NMR spectrum of Pt-BIMA in DMSO-*d*6. A zoom in of the region 5.2 – 9.0 ppm is included.

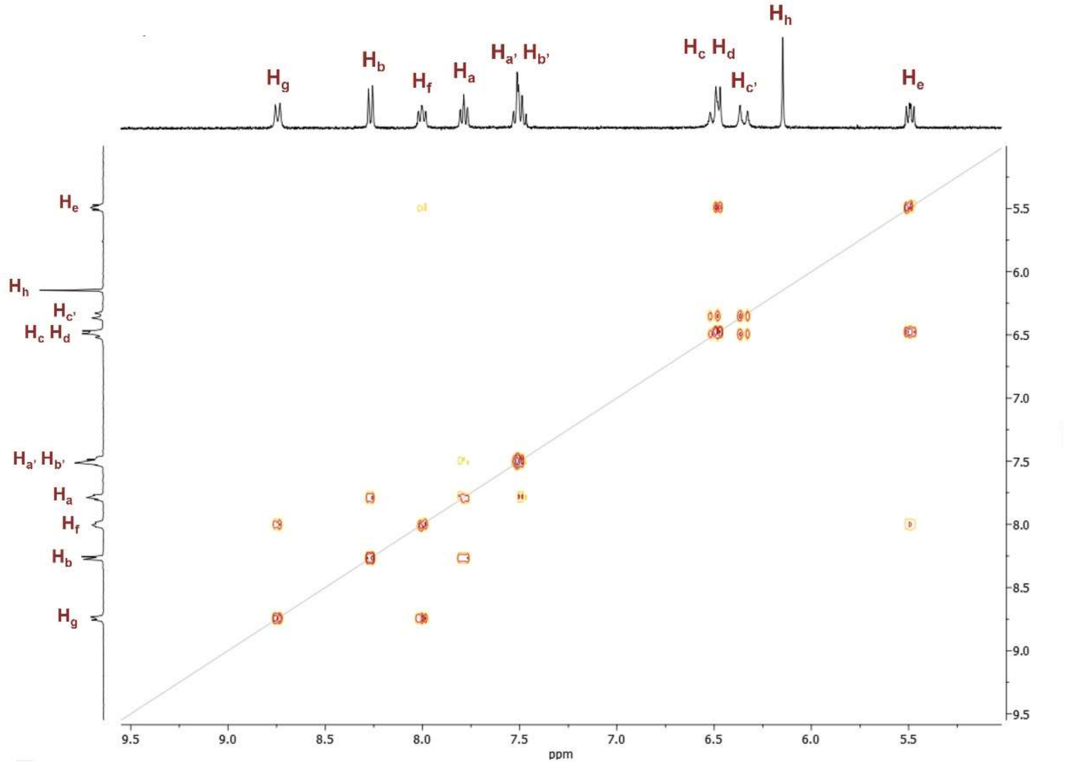

Figure S5. COSY NMR spectrum of Pt-BIMA in DMSO-*d*6 used to assign the ^{1}H peaks (400 MHz).

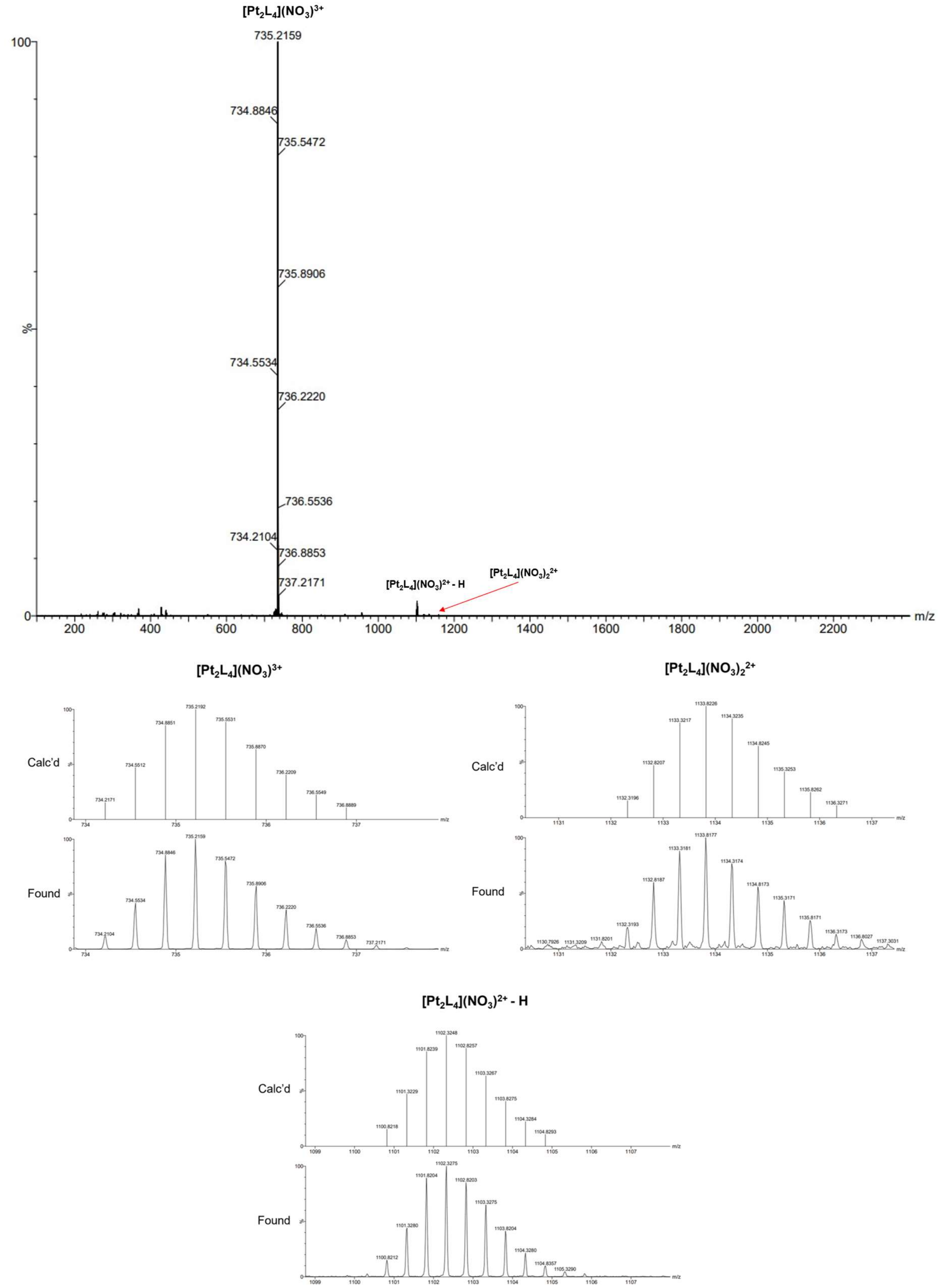

Figure S6. nESI-MS spectrum of Pt-BIMA in acetone. On the right are zoom ins of relevant peaks and the corresponding simulated peaks.

DNA Sequences (5′ to 3′)

DS-21 S1:	CCTTCACGCGAACGTAATCCT
DS-21 S2:	AGGATTACGTTCGCGTGAAGG
4WJ-18 S1:	GCTAGCTGATACGCTACG
4WJ-18 S2:	CGTAGCGTACGTTGGTGC
4WJ-18 S3:	GCACCAACGCGTCACTCC
4WJ-18 S4:	GGAGTGACGTCAGCTAGC
4WJ-22 S1:	GCCTAGCATGATACTGCTACCG
4WJ-22 S2:	CGGTAGCAGTACCGTTGGTGGC
4WJ-22 S3:	GCCACCAACGGCGTCAACTGCC
4WJ-22 S4:	GGCAGTTGACGTCATGCTAGGC
3WJ S1:	CGGAACGGCACTCG
3WJ S2:	CGAGTGCAGCGTGG
3WJ S3:	CCACGCTCGTTCCG
3WJ-18 S1:	GTGGCGAGAGCGACGATC
3WJ-18 S2:	GATCGTCGCAGAGTTGAC
3WJ-18 S3:	GTCAACTCTTCTCGCCAC
mm3WJ S3:	CCACGCACGTTCCG
One-base-bulged 3WJ S1:	CGGAACGAGCACTCG
Two-base-bulged 3WJ S1:	CGGAACGAAGCACTCG
Y fork S1:	CGCACGTACGGAACGGCACTCGCTTGCTCG
Y fork S2:	CGAGCAAGCGAGTGCAGCGTGGATACATGC

Polyacrylamide Gel Electrophoresis (PAGE)

20cm x 20cm large 12% PAGE gels were prepared by mixing 20 mL of 37.5:1 acrylamide/bis-acrylamide with 5 mL of 10X Tris-Boric acid buffer (890 mM, pH 8.3) and 25 mL of Milli-Q water. To this 400 µL of a 10% w/v ammonium persulfate solution in water and 40 µL of TEMED were added to initialise polymerisation. This was then immediately poured between 2 glass plates and a 20-well comb inserted at the top; this was then allowed to set for 1 hr before proceeding.

8.3cm x 7.3 cm mini 12% PAGE gels were prepared by mixing 6 mL of 37.5:1 acrylamide/bis-acrylamide with 1.5 mL of 10X Tris-Boric acid (TB) buffer (890 mM, pH 8.3) and 7.5 mL of Milli-Q water. To this 150 µL of a 10% w/v ammonium persulfate solution in water and 15 µL of TEMED were added to initialise polymerisation. This was then immediately poured between 2 glass plates and a 10-well comb inserted at the top; this was then allowed to set for 1 hr before proceeding.

The gel was then attached to the gel jacket and submerged in 1X TB buffer at the top and bottom. The wells were thoroughly flushed before loading of any sample. Samples were made up to 30 µL containing 1 µM of each DNA strand, 1X buffer (89 mM Tris, 89 mM Boric acid, 10 mM or 50 mM NaCl or 10 mM $MgCl_2$, pH 8.3), and the indicated ratio of complex. DNA, water, and buffer were mixed in solution before addition of the stated ratios of complex. Samples were then centrifuged and incubated at 37° C for 1 hr. 7.5 µL of 50% v/v glycerol was then added to each sample (10% v/v final concentration) and the sample was then centrifuged, mixed, and 10 µL pipetted into the wells on the gel. The gels were run at 140 V for 35 mins (mini gels), 2.5 hrs (large 3WJ gels) or 3 hrs (large 4WJ gels) in 1X TB running buffer. The gel was then removed from the plates and stained using SYBR™ Gold Nucleic Acid Gel Stain (Thermofisher scientific) in 1X TB buffer for at least 30 minutes before imaging on an AlphaImager™ UV transilluminator (Alpha Innotech) with 302 nm excitation.

PAGE Competition Experiments

8.3cm x 7.3 cm mini 12% PAGE gels were prepared as described previously in this document. The gel was then attached to the gel jacket and submerged in 1X TB buffer at the top and bottom. The wells were thoroughly flushed before loading of any sample. Samples were made up to 20 µL containing 2

μM of fluorescently labelled DNA (either 3WJ or 4WJ-18 with a 5′ FAM label on strand S1), 1X TBN buffer (89 mM Tris, 89 mM Boric acid, 50 mM NaCl, pH 8.3), 2 μM Pt-BIMA, and the indicated ratio of competitor DNA. DNA, water, and buffer were mixed in solution before addition of the stated ratios of complex. Samples were then centrifuged and incubated at 37° C for 1 hr. 5 μL of 50% v/v glycerol was then added to each sample (10% v/v final concentration) and the sample subsequently centrifuged, mixed, and 8 μL pipetted into the wells on the gel. The gels were run at 140 V for 40 mins in 1X TB running buffer. The gel was then removed from the plates and rinsed in deionised water for 5 minutes before imaging on an AlphaImager™ UV transilluminator (Alpha Innotech) with 302 nm excitation. ImageJ was used to quantify the intensity of the gel bands.[7] The intensities of the 3WJ/4WJ bands were measured as a fraction of the total lane intensity (ssDNA band intensity + 3WJ band intensity) and normalised to the lane containing no competitor DNA. All competitor ratios were measured as the average of 3 independent samples.

Fluorescence Spectroscopy

Fluorescence spectra were recorded on a Cary Eclipse Fluorescence Spectrophotometer (Agilent Technologies, Inc.). Pt-BIMA was used from a 1 mM stock concentration in 50% aqueous DMSO. Pd-BIMA was used from a 1 mM stock in pure DMSO. Samples were made up to 1 mL and pipetted into a 1 cm path length, quartz fluorescence cuvette. Data was plotted in MATLAB.

Excitation spectra of Pd-BIMA and Pt-BIMA alone were recorded by monitoring the emission at 425 nm and 426 nm respectively. Emission spectra were recorded with an excitation wavelength of 372 nm or 376 nm respectively. Excitation spectra of the 4WJ-FAM samples were recorded by monitoring the fluorescence intensity at 520 nm using a 475 nm cutoff filter. Emission spectra were recorded with an excitation wavelength of 375 nm. Samples contained 5 μM FAM-labelled 4WJ S1 (FAM1, FAM7 or FAM11), 5 μM 4WJ S2, 4WJ S3 and 4WJ S4, 10 mM HEPES, 50 mM NaOAc and either 5 μM Pt-BIMA or an equivalent volume of 50% aqueous DMSO.

Spectral overlap between the absorbance spectrum of the 4WJ-FAMs and the emission of Pt-BIMA was calculated using the a|e - UV-Vis-IR Spectral Software (FluorTools, www.fluortools.com). The spectral overlap with 4WJ-FAM1 is 4.872 x 10^{14} nm^4 M^{-1} cm^{-1}, with 4WJ-FAM7 is 4.454 x 10^{14} nm^4 M^{-1} cm^{-1} and with 4WJ-FAM11 is 5.478 x 10^{14} nm^4 M^{-1} cm^{-1}.

UV-Visible Spectroscopy

Samples for obtaining the UV-VIS spectra of the compounds in water were prepared by diluting a 1 mM working stock in DMSO to 20 μM with water. These same samples were then left in the cuvettes and recorded each day for 7 days, and the once again after 14 days. Absorbance was recorded between 200-800 nm (1 nm bandwidth, 600 nm/min) in a Cary5000 UV-Vis-NIR Spectrophotometer (Agilent Technologies, Inc.) with a multi-cell holder. Spectra for the FAM-labelled 4WJ samples used in the fluorescence experiments were obtained after transferring the sample into a 1 cm path length, masked quartz cuvette. Absorbance was recorded between 220-600 nm (1 nm bandwidth, 600 nm/min) in a Cary60 UV-VIS Spectrophotometer (Agilent Technologies, Inc.). In all cases, each spectrum was zeroed and a baseline recorded for each condition. Data was plotted in MATLAB.

UV Melting

The stability of 4WJ, 3WJ, Y fork and dsDNA in the absence and presence of Pt-BIMA was monitored by measuring the absorbance at 260 nm with increasing temperature. 4WJ-22 and 3WJ-18 were chosen as the junctions for this study as they form spontaneously in the absence of compound. Each sample contained 1 μM of each oligo, 1 μM of Pt-BIMA, 1% DMSO, 10 mM sodium cacodylate (pH 7.4) and 50 mM NaOAc (chosen instead of NaCl to avoid issues with chloride coordination to the Pt). Control samples were also prepared containing all components except Pt-BIMA. Samples were made up to 1500 μL volume and pipetted into 1500μL masked quartz cuvettes with 1 cm path length and the cuvette then stoppered. The measurements were carried out on a Cary5000 UV-Vis-NIR spectrophotometer (bandwidth, 1 nm; average time 1 s; heating rate, 0.5˚ C min^{-1}; measurement interval, 0.5 ˚C) equipped with a multi-cell holder and peltier temperature controller. Data was collected in triplicate for each condition and a blank sample was ran concurrently allowing for immediate baseline correction. The data was normalised and plotted in MATLAB, and the melting temperature (T_m) determined as the temperature at the derivative maximum. The final melting temperature was then reported as the average

of the three runs with standard deviation error.

Microscale Thermophoresis (MST)
A 1 mM stock solution of Pt-BIMA in 50% aqueous DMSO was diluted to either 100 µM or 10 µM in buffer. The buffer used was HEPES 10 mM, 50 mM NaOAc, 0.1% Tween, as previously reported for use with DNA junctions in MST. [8] A 1 mM stock solution of Au pillarplex in water was diluted to 10 µM in buffer. Samples were prepared by making a serial dilution of complex (Pt-BIMA or Au pillarplex) in buffer and mixing with either 40 nM FAM-labelled 4WJ-22, 3WJ-18 or 80 nM DS-21. In competition experiments, 20 nM (1 equivalent with respect to the labelled 4WJ) of the non-labelled competitor DNA (3WJ-18 or DS-21) was contained in the buffer. In competition titrations, competitor DNA (DS-21, 3WJ, 4WJ) were serially diluted and mixed with a mixture containing 40 nM FAM-4WJ and 160 nM Pt-BIMA. Samples were incubated at room temperature for at least 15 minutes before loading into Monolith standard capillary tubes and the tubes then placed into a Monolith NT.115 (Nanotemper Technologies). MST experiments were run at room temperature (22 °C) using 60% excitation power (blue laser) and medium MST power. Each experiment was done in triplicate. The data was analysed and plotted using MO.Affinity Analysis software (Nanotemper Technologies), fitting the data to a k_d model.

Time-Correlated Single Photon Counting (TCSPC)
TCSPC data was collected using a custom built time correlated single photon counting spectrometer. Solutions were irradiated with an Edinburgh Instruments EPL375 diode laser (375 nm, 10 kHz, 65 ps bandwidth), with luminescence collected perpendicular to the excitation axis. The luminescence is collected using a Nikon 4x, 17.2 mm WD microscope objective, and the collimated luminescence is passed through a 375 nm longpass filter to remove the influence of laser scatter from the resulting emission. Luminescence is collected by an ID Quantique single photon counting detector (ID100). Photon counting events are digitally processed and binned using a Swabian Time Tagger 20 (20 ps time resolution) and are converted into a time dependent histogram using the commercial Swabian Instruments software suite. Lifetimes are analysed by iterative reconvolution fitting within a slightly modified version of the AnalyseItRe software package provided by Smith et al,[9] with an IRF with FWHM of 216 ps.

Single Molecule FRET
Solution-based smFRET experiments were performed on the smfBox.[10] Briefly, FRET labelled 4WJs (Atto550 and Atto647N) were diluted to 10 pM in 10 mM Tris-Acetate (containing either 50 mM NaOAc or 50 mM $Mg(OAc)_2$) and observed on a coverslip using Alternating Laser Excitation (515 nm 50 µs, 635 nm 50 µs), the fluorescence for each dye being collected through the same objective (Olympus UPLSAPO ×60 NA = 1.35 oil immersion), focused onto a pin hole (20 µm) and then split according to wavelength by a dichroic mirror (Chroma NC395323—T640lpxr) onto two separate avalanche photodiodes (SPCM-AQRH-14, Excilitas). As individual 4WJs diffuse through the excitation volume, bursts of photons are potentially observed, and photon counts in three channels (DD, DA, AA) potentially used to calculate the FRET efficiency and stoichiometry (see ref. 9). Pt-BIMA was added at 10 µM to ensure saturated binding, however strong fluorescence quenching of the fluorophore by the excess compound prevented detailed analysis.

MOLECULAR DYNAMICS SIMULATIONS

Parameterisation of Pd-BIMA and Pt-BIMA
Parameters for the coordination bonds were calculated using the MCPB.py pipeline with Gaussian16 at the ωB97XD/DEF2-SVP level of theory to include dispersion, with ECP for Pd and Pt. [11-12] The output coordinate and topology files were converted to GROMACS format using ParmEd (https://github.com/ParmEd/ParmEd). Coordinates for the mirror image enantiomers were generated using the invert chirality function in Avogadro. [13]

Parameterisation of DNA

The PDB file for the 25mer B-DNA consisting of 2 strands (A_{25} and T_{25}) was generated using NAB (nucleic acid builder) in AmberTools.[14] The 3WJ structure was adapted from PDB 1F44,[15] as described previously.[16] The 4WJ structure was taken from the 1XNS PDB crystal structure,[17] and adapted as described previously.[16] The "true" 4WJ (in which all base pairs are intact from the beginning) was taken from a snapshot of an MD simulation we previously reported containing an organometallic pillarplex in the cavity; [16] the pillarplex was removed leaving the DNA alone. Similarly, the closed 4WJ structure was taken from a snapshot of an MD simulation of the free 4WJ (i.e. with no compound). All DNA was parameterised using the AMBER forcefield parmbsc1.[18]

Simulations

In simulations with the 1XNS 4WJ, the compounds were placed outside but close to the open cavity of the structure. (When placed further away, the compound would not enter the cavity before the 4WJ closed). In simulations with the true 4WJ, the compounds were placed directly inside the open cavity. In simulations with the closed 4WJ, the compounds were placed close to the centre of the 4WJ. In all simulations of 3WJ, the compounds were similarly placed outside but close to the cavity. The two-bases-bulged 3WJ starting structure was taken from a snapshot a simulation of the 3WJ with Pt-BIMA, in which the 3WJ rearranged itself into the two-base-bulged 3WJ. In further simulations using this starting structure, compounds were placed directly in the cavity. The one-base-bulged 3WJ structure was generated by deleting one of the unpaired bases in the two-based-bulged structure and manually moving the strands closer together in PyMOL, and then energy minimising the structure in GROMACS. In all simulations with the B-DNA, multiple compounds were placed within 1 nm distance of the DNA. In all simulations, DNA was placed with the compounds in a dodecahedral box with periodic boundary conditions. MD preparation steps were carried out using GROMACS software as described previously[16, 19-20]: All systems were solvated in water using the TIP3P model and neutralised with Na^+ ions. Additional Na^+ and Cl^- ions were added to reach a NaCl concentration of 50 mM. Initial minimisation was carried to at least 500 kJ/mol/nm or 50000 steps followed by heating and NVT equilibration for 1000 ps using V-rescale modified Berendsen thermostat, coupling the cylinder with the DNA at 310 K. All simulations use 2 fs time step and Parrinello-Rahman pressure coupling and PME electrostatics at 1.0 nm cutoff. All simulations were run on the BlueBEAR cluster at U. Birmingham using GROMACS software. After the simulations had finished, the trajectories were processed in GROMACS to remove periodic boundary conditions, translations and rotations, and visualised in PyMOL.[21] RMSD plots were calculated in GROMACS and representative plots are included in Figure S13.

SUPPLEMENTARY DATA

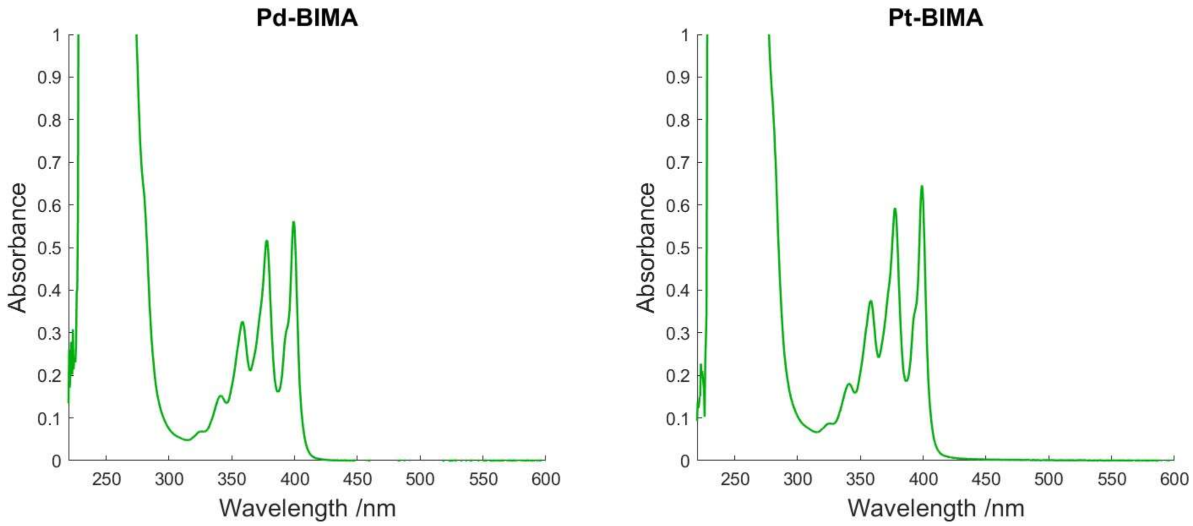

Figure S7. Absorbance spectra of Pd-BIMA (left) and Pt-BIMA (right), 20 μM in water (2% or 1% DMSO respectively), recorded at room temperature.

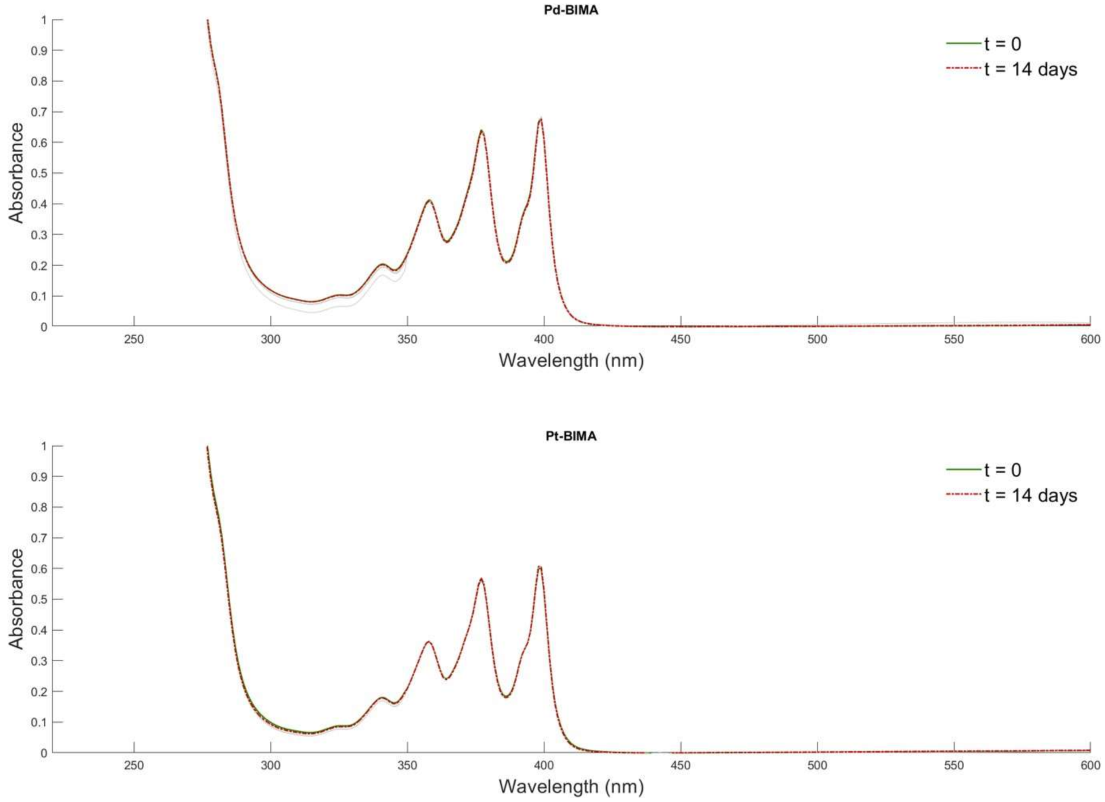

Figure S8. Absorbance spectra of Pd-BIMA (top) and Pt-BIMA (bottom) in water at 20 μM (2% DMSO) measured over time at room temperature. The green line (hidden due to overlap with other lines) shows the absorbance at t = 0 and the dashed red line shows the absorbance at t=14 days. Grey lines show intermediate time points. It is clear there is little to no change in the absorbance spectra over time and so it can be concluded that the compounds are stable in this solution, under these conditions.

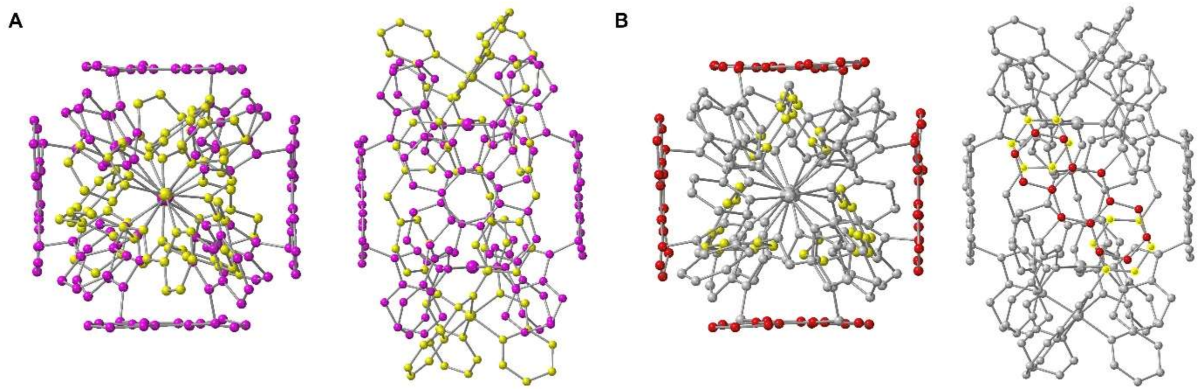

Figure S9. A) Overlay of the crystal structures of the iron(II) triple-stranded cylinder (a known 3WJ binder, shown in yellow) and Pd-BIMA (shown in purple) superimposed and shown end on (left) and side on (middle). B) The DNA-binding surfaces of Pd-BIMA (red) and cylinder (yellow) are highlighted illustrating the tetragonal disposition of the Pd-BIMA anthracenes (suited to 4WJ) and the trigonal arrangement of the cylinder spacer surfaces (perfect for 3WJ). Hydrogens have been omitted for clarity.

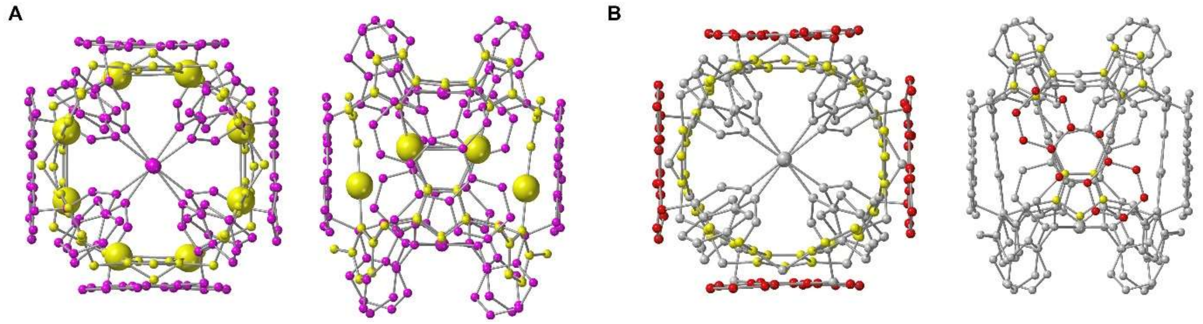

Figure S10. A) Overlay of the crystal structures of the organometallic gold pillarplex (a known 4WJ binder, shown in yellow) and Pd-BIMA (shown in purple) superimposed and shown end on (left) and side on (right). B) The DNA-binding surfaces of Pd-BIMA (red) and pillarplex (yellow) are highlighted illustrating the larger radius of Pd-BIMA, as well as the more central positioning of the aromatic moieties on its surface. Hydrogens have been omitted for clarity.

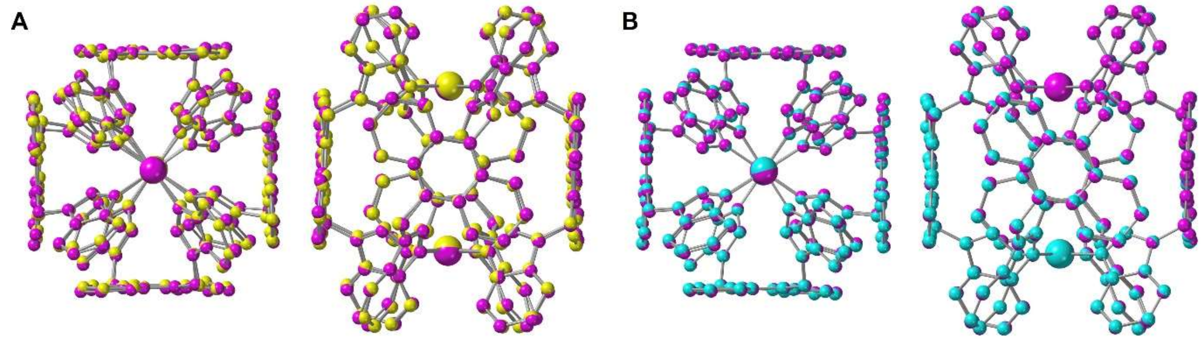

Figure S11. A) Overlay of the DFT geometry optimisation (pink) and the reported crystal structure (yellow) of Pd-BIMA. B) Overlay of the DFT geometry optimisation outputs of Pd-BIMA (pink) and Pt-BIMA (cyan). The structures are essentially isostructural. Hydrogens have been omitted for clarity.

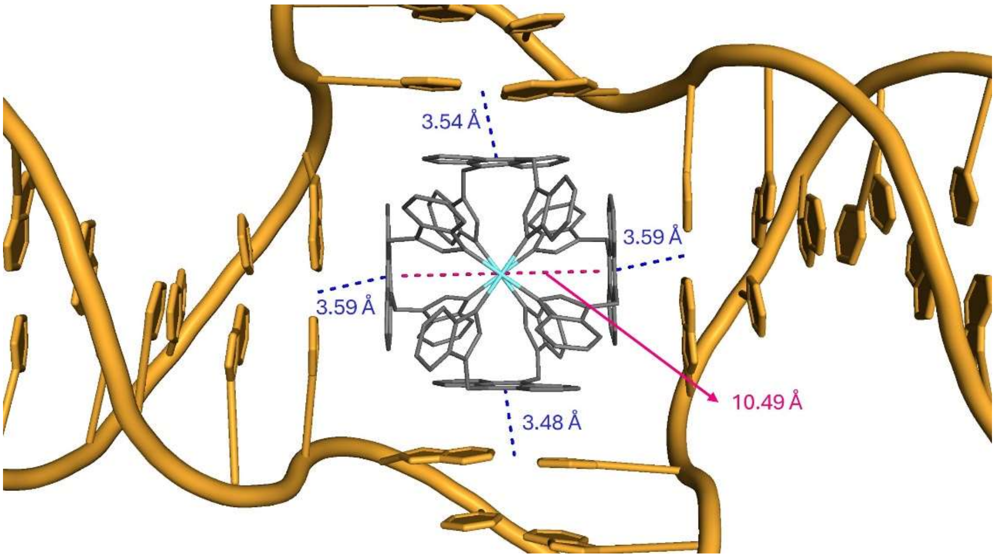

Figure S12. Zoom in of an MD snapshot from a simulation of the Pt-BIMA M enantiomer bound in the 4WJ cavity. The dashed lines represent the anthracene-anthracene distance (pink) and the anthracene-base pair distances (blue). The optimal π-stacking distance is expected to be 3.5 Å - the compound is able to achieve this on all four sides, highlighting the high complementarity between the dimensions of the compound and the 4WJ cavity. Hydrogens have been omitted for clarity.

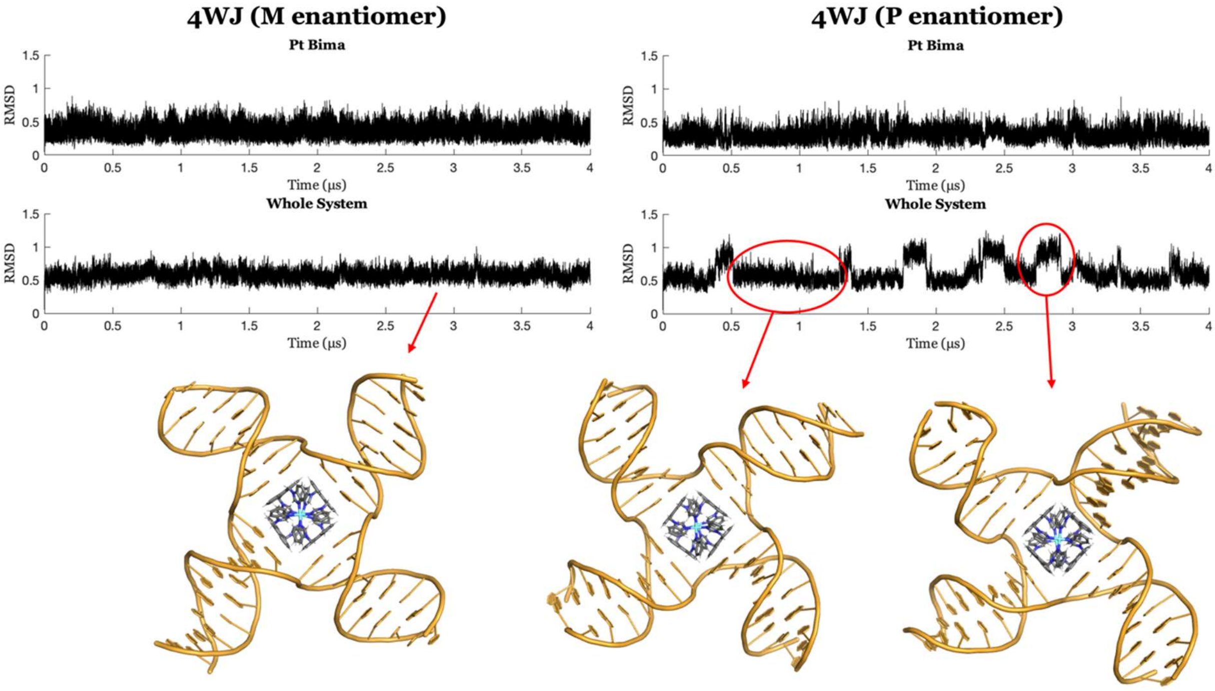

Figure S13. RMSD plots for representative simulations of both enantiomers of Pt-BIMA inside the 4WJ. The spikes in the P enantiomer plot represent large dynamic changes in the 4WJ conformation. Shown underneath are structure snapshots corresponding to the region on the RMSD plot.

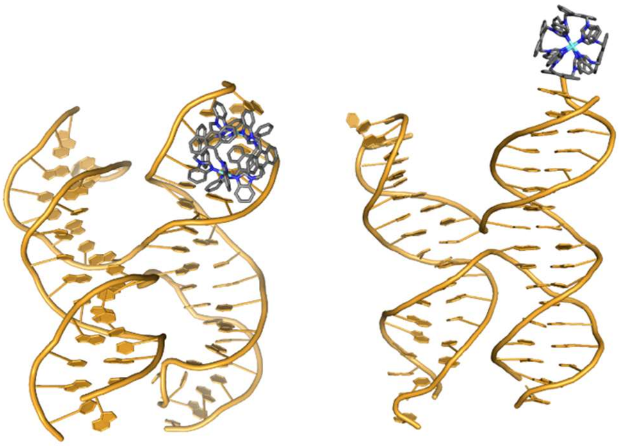

Figure S14. MD snapshots of the P enantiomer of Pt-BIMA binding to the closed X-stacked 4WJ in the minor groove of a duplex arm (left) and at a duplex arm terminus. Hydrogens are omitted for clarity.

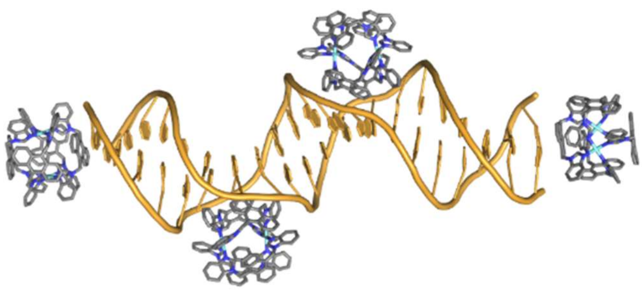

Figure S15. MD snapshot of a 2 μs simulation of a 25mer polyAT B-DNA duplex with 4 Pt-BIMA compounds (racemic mix). Binding is seen at the duplex termini, where the anthracene units π-stack with the terminal base pairs; and transiently in the minor groove, where the compounds flatten the conformation of the helix and are seen to slide along the groove. Hydrogens are omitted for clarity.

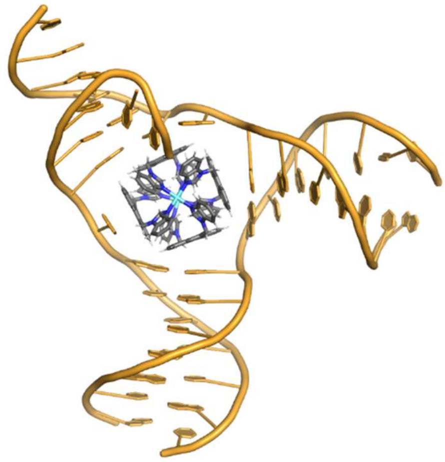

Figure S16. MD snapshot of the P enantiomer inside the 3WJ, exhibiting partial melting of one duplex arm, which allows one strand to fold back towards the central cavity and for a terminal nucleotide to interact with Pt-BIMA. Hydrogens are omitted for clarity.

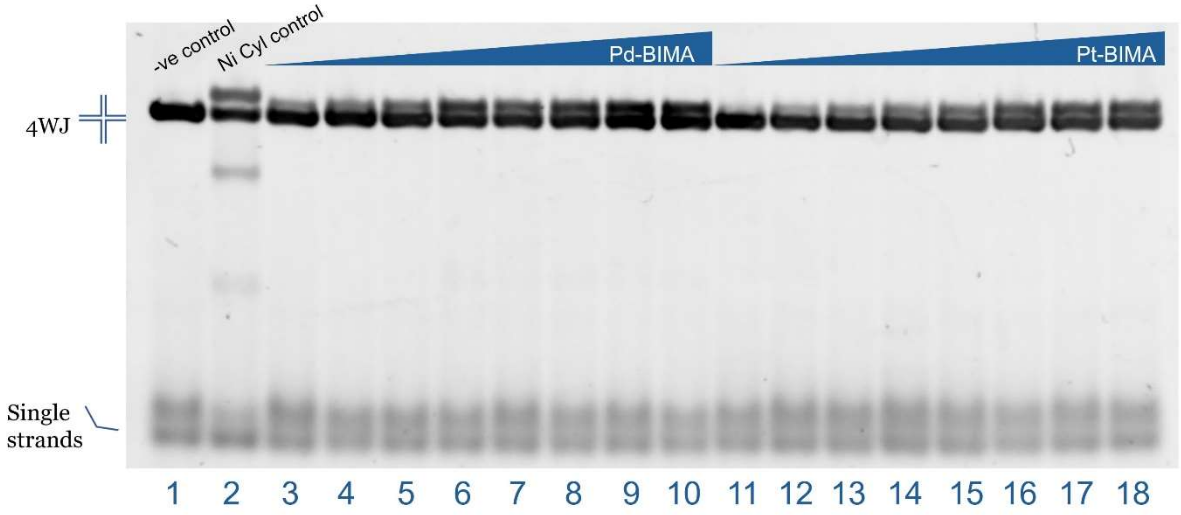

Figure S17. PAGE gels showing binding of Pd-BIMA and Pt-BIMA to a 4WJ-22 (which forms spontaneously) in 50 mM NaCl. Gels contains DNA alone (lane 1), DNA + Ni Cylinder (lane 2), DNA + 0.25, 0.5, 1, 1.5, 2, 3, 4, 5 equiv. Pd-BIMA (lanes 3-10) and DNA + 0.25, 0.5, 1, 1.5, 2, 3, 4, 5 equiv. Pt-BIMA (lanes 11-18). Gel samples were made up in 1X TB (89mM Tris base, 89mM boric acid) and 50Mm NaCl and incubated at 37 °C for 1 hour.

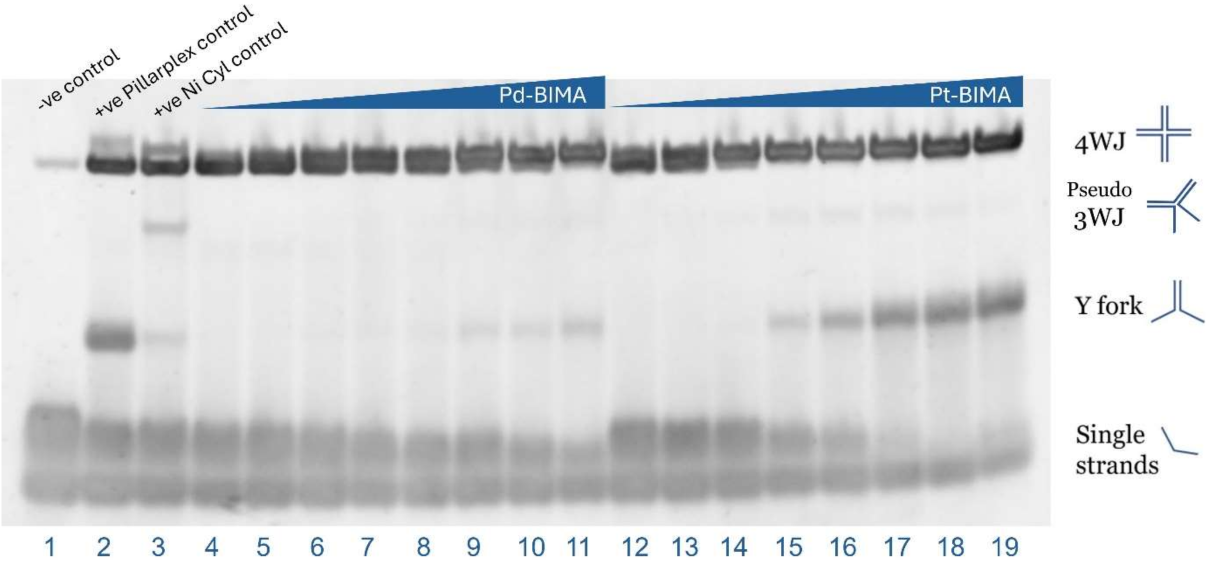

Figure S18. PAGE gel showing binding of Pd-BIMA and Pt-BIMA to 4WJ-22 in 10 mM NaCl. Gels contains DNA alone (lane 1), DNA + Ni Cylinder (lane 2), DNA + 0.25, 0.5, 1, 1.5, 2, 3, 4, 5 equiv. Pd-BIMA (lanes 3-10) and DNA + 0.25, 0.5, 1, 1.5, 2, 3, 4, 5 equiv. Pt-BIMA (lanes 11-18). Gel samples were made up in 1X TB (89mM Tris base, 89mM boric acid) and 10Mm NaCl and incubated at 37 °C for 1 hour.

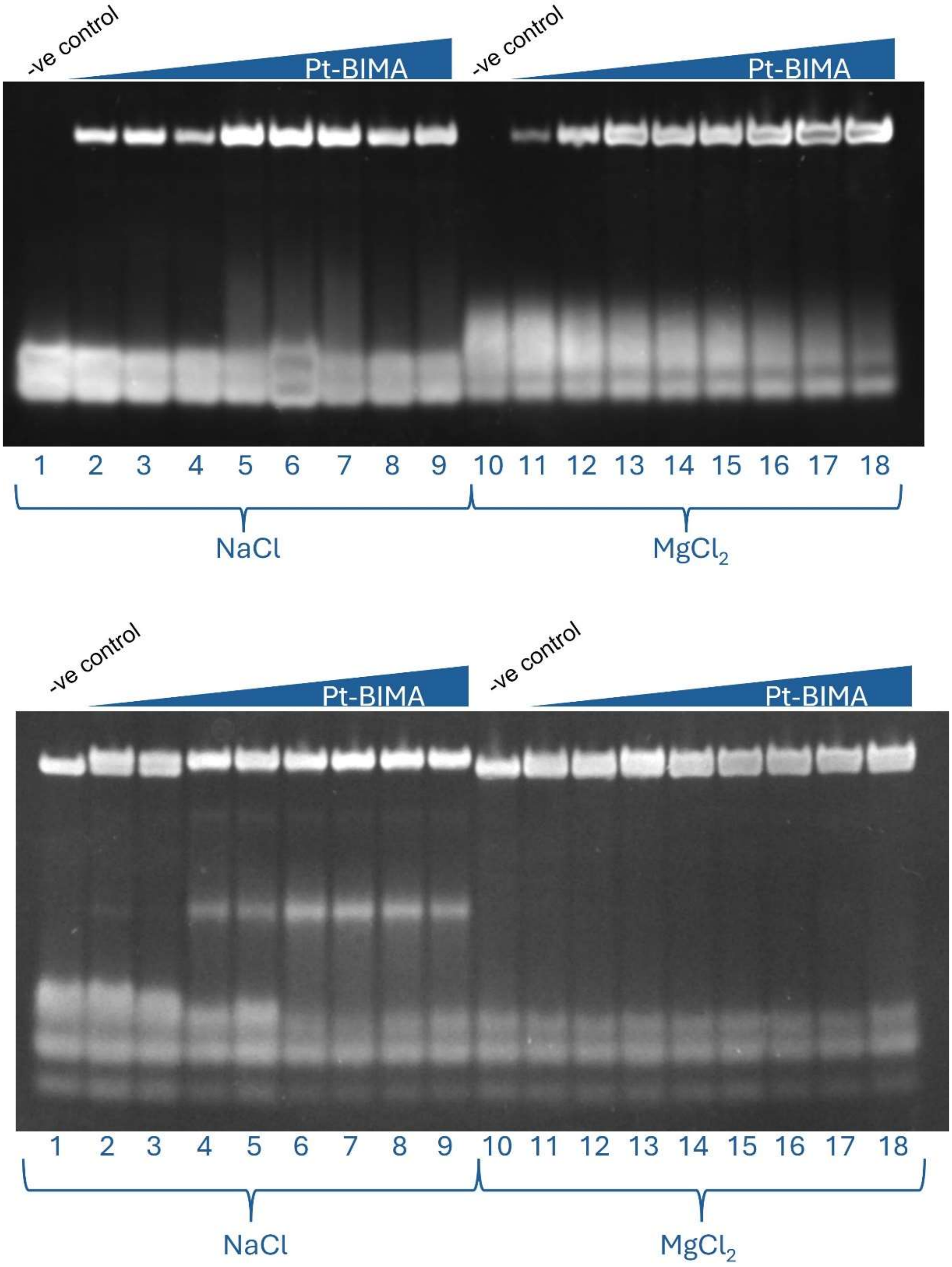

Figure S19. PAGE gels of 4WJ-18 (top) and 4WJ-22 (bottom) with increasing concentrations of Pt-BIMA (0, 0.25, 0.5, 1, 1.5, 2, 3, 4, 5 equiv.) in the presence of 10 mM NaCl (lanes 1-9) and 10mM $MgCl_2$ (lanes 10-18).

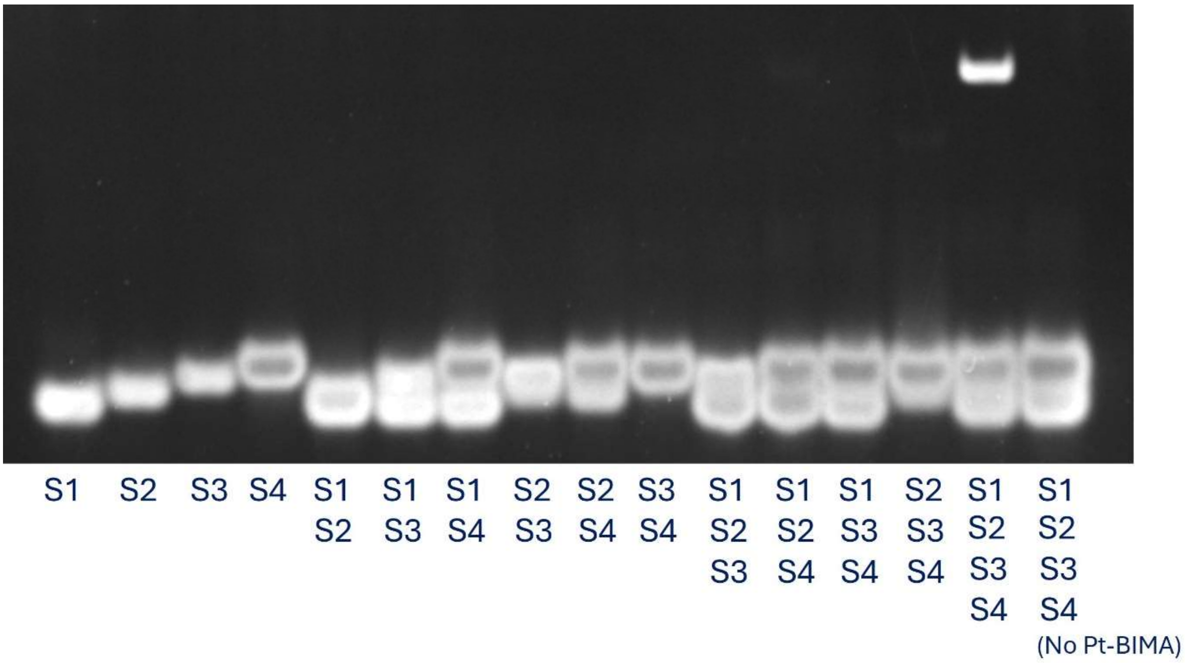

Figure S20. PAGE gel showing the interaction of Pt-BIMA with all combinations of the 4WJ-18 strands. All lanes contain 1 equiv. Pt-BIMA (except the last lane). Samples prepared in 1X TB buffer, 50 mM NaCl.

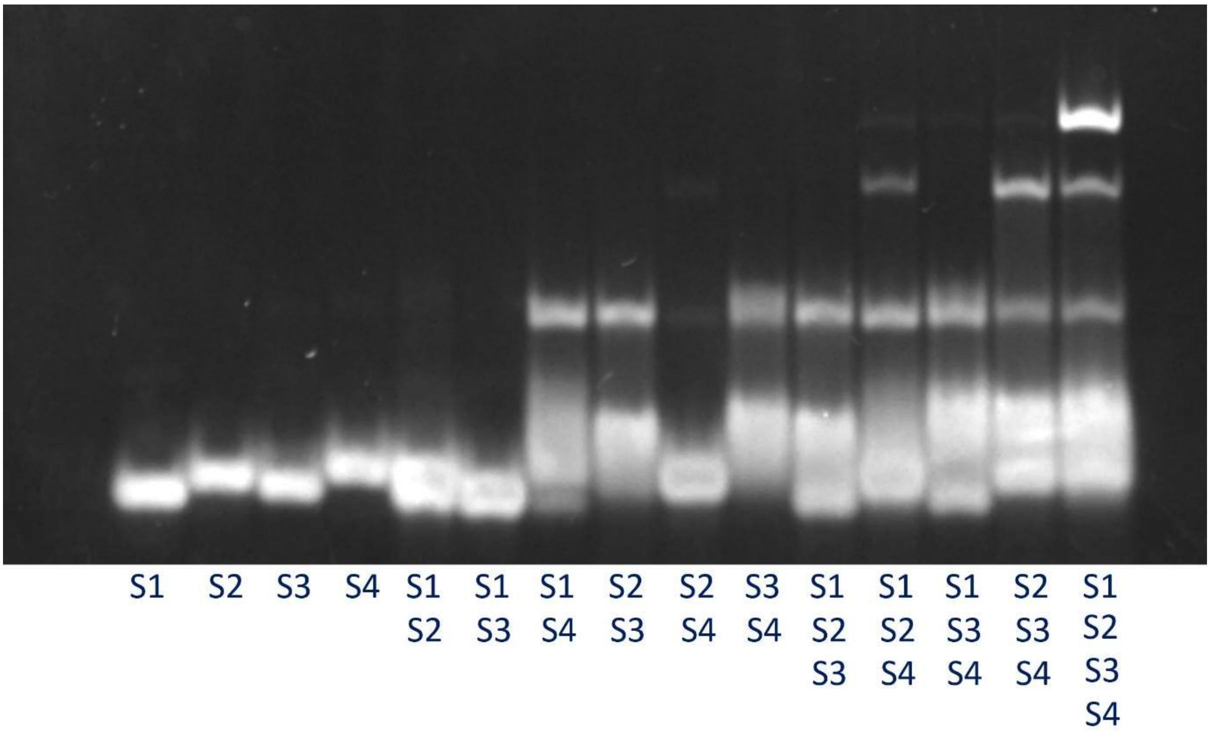

Figure S21. PAGE gel showing the interaction of Pt-BIMA with all combinations of 4WJ-22 strands. All lanes contain 1 equiv. Pt-BIMA. Samples prepared in 1X TB buffer, 10 mM NaCl.

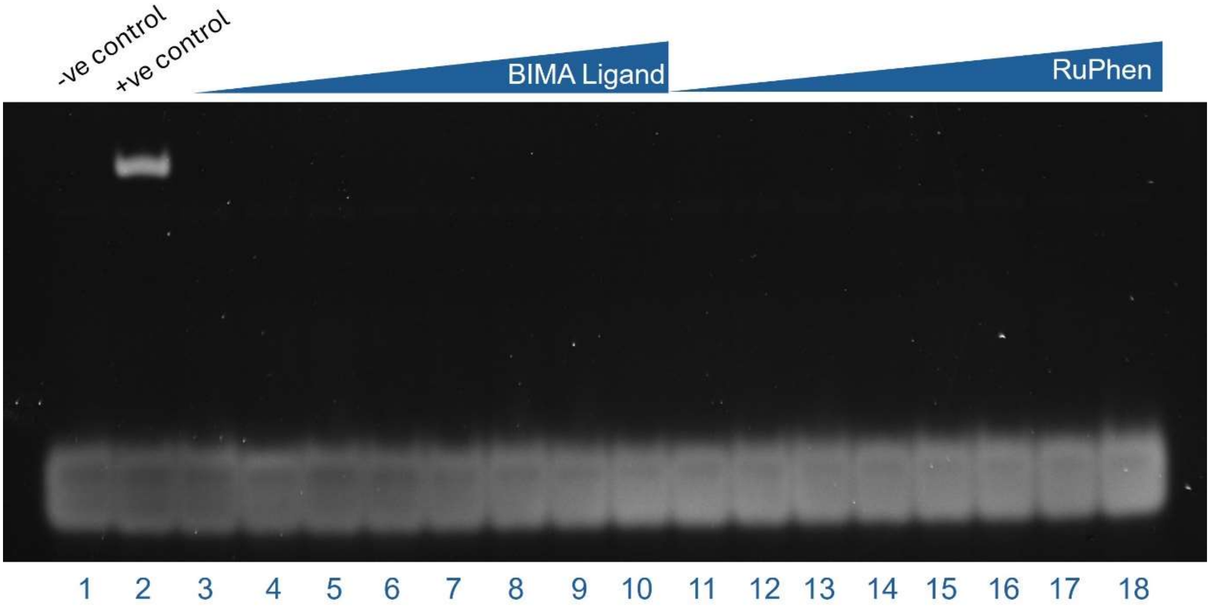

Figure S22. PAGE control gels showing that the BIMA ligand and $[Ru(phen)_3]Cl_2$ (used here as a metal complex that should be a negative control) do not induce the formation of 4WJ-18 (A) or bind to the 4WJ-22 (B). In both gels, samples are: DNA only (lane 1), DNA + Pd-BIMA (lane 2), DNA + 0.25, 0.5, 1, 1.5, 2, 3, 4, 5 equiv. BIMA ligand (lanes 3-10), DNA + 0.25, 0.5, 1, 1.5, 2, 3, 4, 5 equiv. $[Ru(phen)_3]Cl_2$ (lanes 11-18).

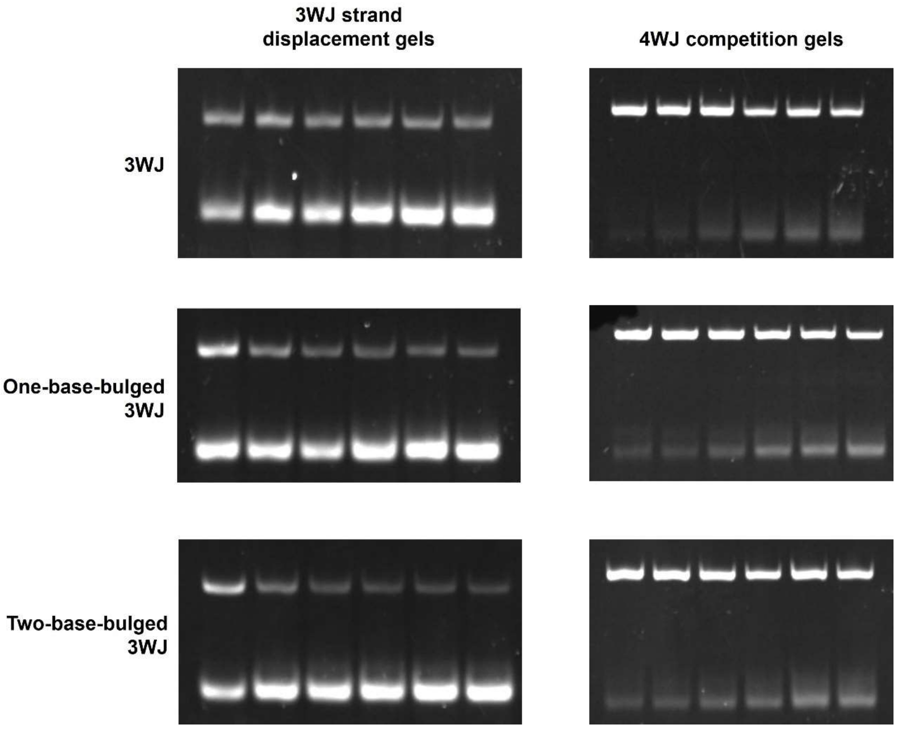

Figure S23. Representative images of gels from each PAGE strand displacement or competition experiment (one representative gel per experiment). From left to right are lanes containing 2 µM FAM-labelled (on strand S1) 3WJ and 2 µM Pt-BIMA with increasing concentrations of (non-labelled) competitor S1 strands (0, 0.25, 0.5, 1, 1.5, 2, 3, 4, equivalents).

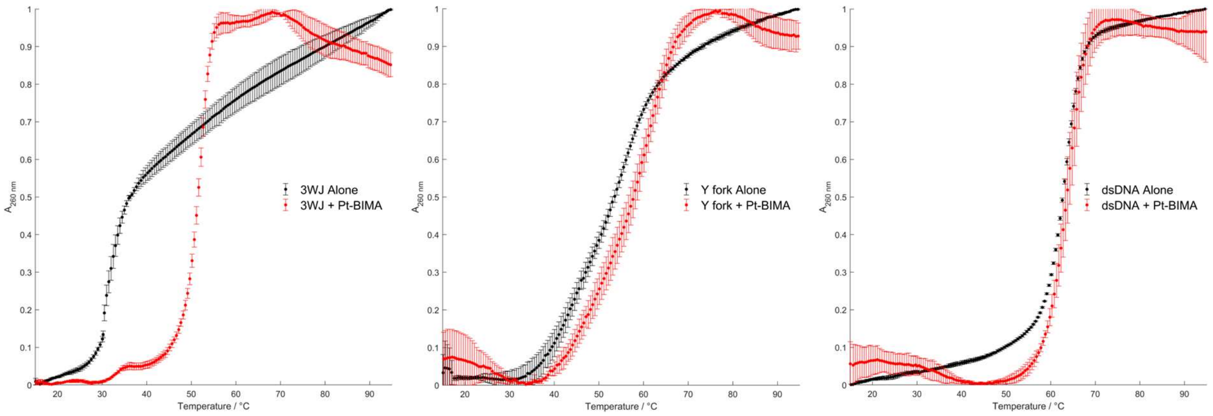

Figure S24. UV melting curves of 3WJ-18, Y fork and dsDNA with (red) and without (black) 1 equivalent of Pt-BIMA (10 mM Na cacodylate, 50 mM NaOAc, 0.05% DMSO). Each curve was normalised to its highest and lowest absorbance measurements. Error bars represent that standard deviation of 3 biological replicates. The observed melting temperature of the 3WJ was 31.1 ± 0.1 °C alone and 52.2 ± 0.1 °C with Pt-BIMA (ΔT_m = 21.1 ± 0.1 °C). The observed melting temperature of the Y fork was 55.5 ± 0.6 °C alone and 60.9 ± 0.5 °C with Pt-BIMA (ΔT_m = 5.4 ± 1.1 °C). The observed melting temperature of the dsDNA was 63.4 ± 0.1 °C alone and 64.1 ± 0.8 °C (ΔT_m = 0.7 ± 0.9 °C). The melting temperatures for the 3WJ and 4WJ with Pt-BIMA are near identical and thus may represent the temperature of dissociation of the compound from a junction cavity (at which point, both junctions fall apart completely as it is well above the T_m of the free DNAs). It is important to note that, whilst these experiments confirm binding of Pt-BIMA to, and stabilisation of, the 4WJ, 3WJ and Y fork, the ΔT_m values obtained are not directly comparable between the DNA structures as they each contain a different number of strands, different strand lengths and different sequences. Alternative techniques (such as MST used in this work) are required to obtain a direct comparison of the thermodynamic parameters of the binding.

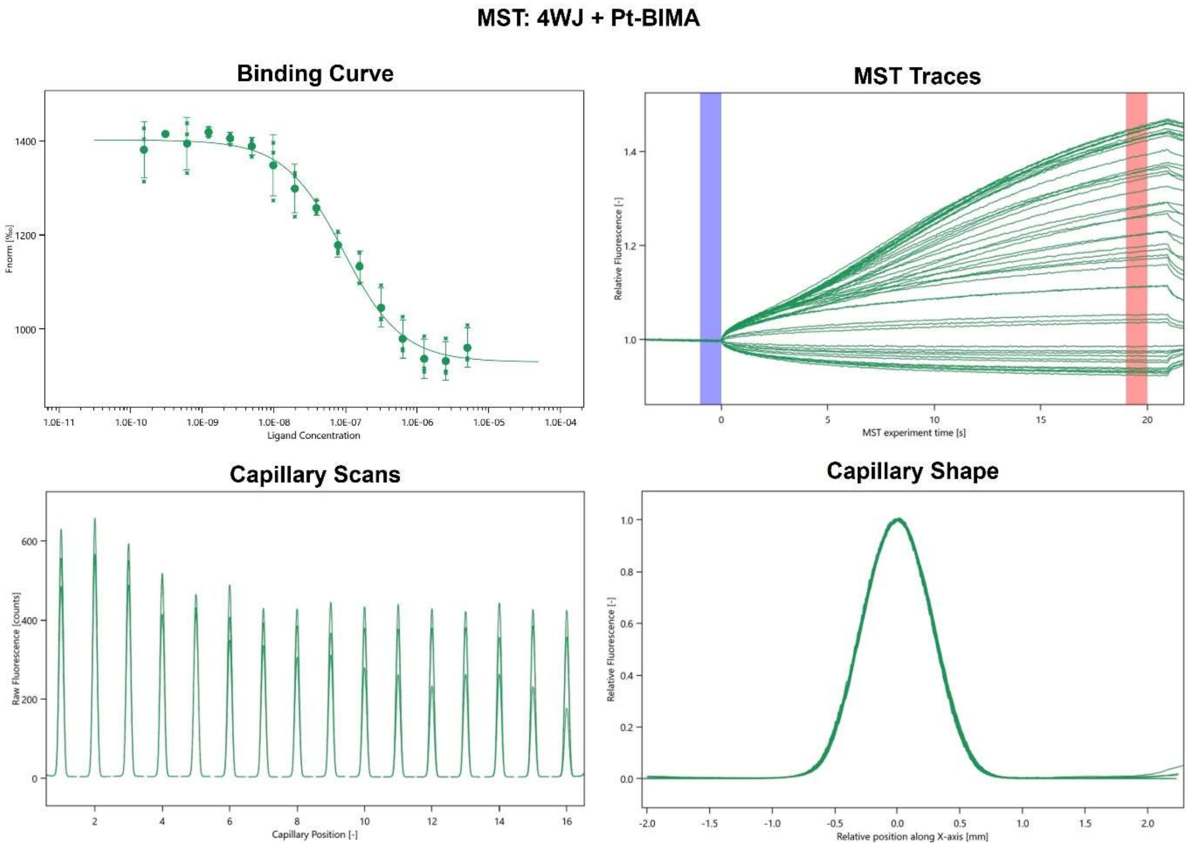

Figure S25. Top left: Binding curve obtained from MST experiments with Pt-BIMA and 4WJ. Pt-BIMA was serially diluted from 5 µM to 153 pM and mixed with 20 nM FAM-4WJ. Each data point is shown as the average of 3 repeats (this graph is also shown in the main paper figure 4B). Top right: MST traces for all samples (all repeats). The red shading represents the time range in which the average fluorescence intensity was measured to obtain the binding curve (19-20 s after IR irradiation) and the blue shading represents the initial fluorescence, to which the measurements are normalised. Bottom left: Fluorescence intensity scans of each capillary for each MST repeat prior to IR irradiation. Bottom right: Shape of the fluorescence curves of each capillary across each repeat prior to IR irradiation. Samples contained 10 mM HEPES, 50 mM NaOAc, 0.1% Tween-20, 0.25% DMSO. $k_d = 8.27 \pm 1.11 \times 10^{-8}$ M.

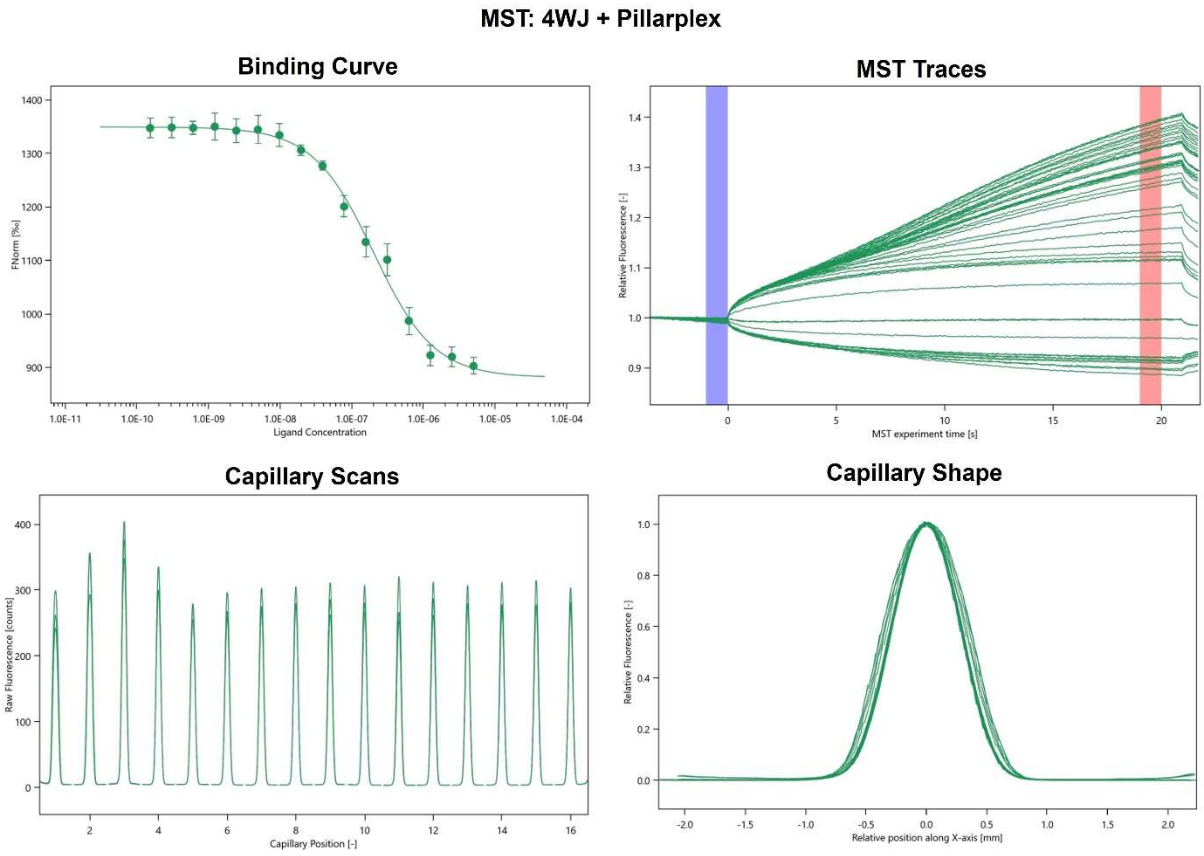

Figure S26. Top left: Binding curve obtained from MST experiments with Au pillarplex and 4WJ. Au pillarplex was serially diluted from 5 µM to 153 pM and mixed with 20 nM FAM-4WJ. Each data point is shown as the average of 3 repeats. Top right: MST traces for all samples (all repeats). The red shading represents the time range in which the average fluorescence intensity was measured to obtain the binding curve (19-20 s after IR irradiation) and the blue shading represents the initial fluorescence, to which the measurements are normalised. Bottom left: Fluorescence intensity scans of each capillary for each MST repeat prior to IR irradiation. Bottom right: Shape of the fluorescence curves of each capillary across each repeat prior to IR irradiation. Samples contained 10 mM HEPES, 50 mM NaOAc, 0.1% Tween-20. $k_d = 1.91 \pm 0.20 \times 10^{-7}$ M.

MST: dsDNA + Pt-BIMA

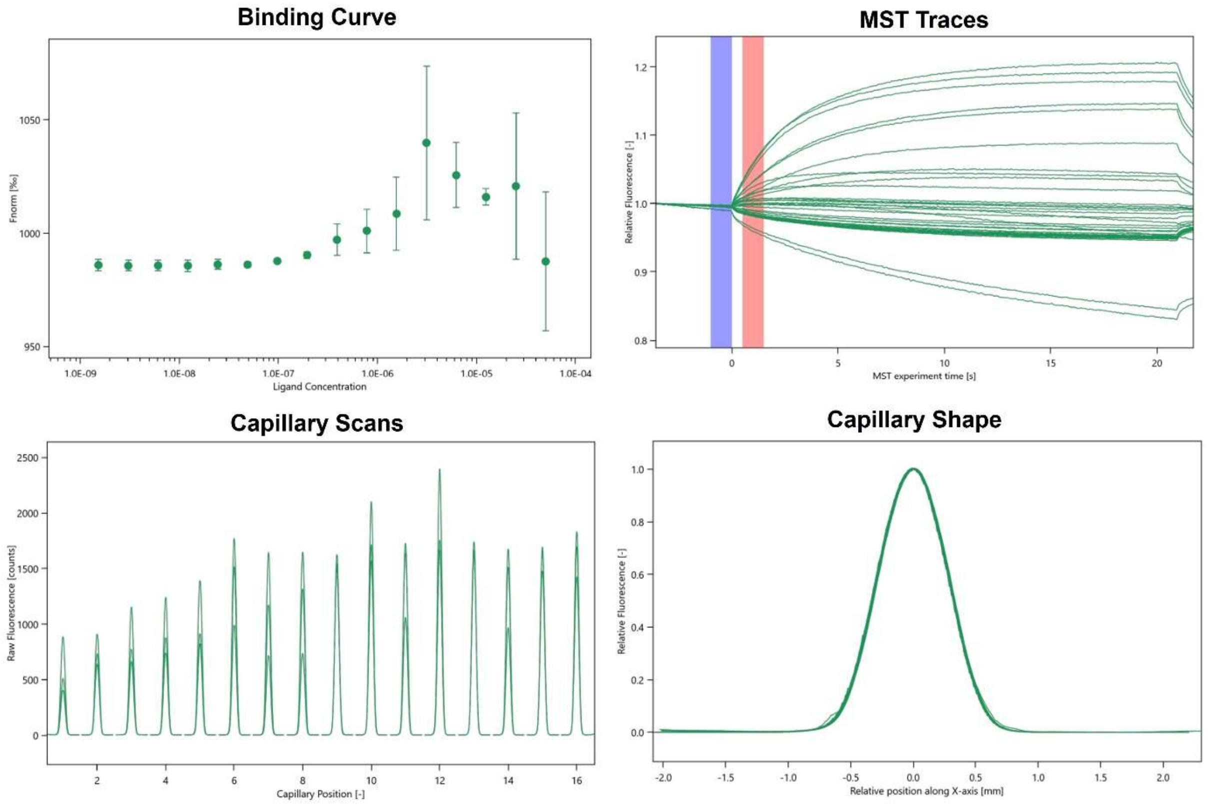

Figure S27. Top left: Binding curve obtained from MST experiments with Pt-BIMA and dsDNA. Pt-BIMA was serially diluted from 50 µM to 1.53 nM and mixed with 40 nM FAM-dsDNA. Each data point is shown as the average of 3 repeats. The lack of a sigmoidal curve indicates that there is no binding in this concentration range. Top right: MST traces for all samples (all repeats). The red shading represents the time range in which the average fluorescence intensity was measured to obtain the binding curve (1.5-2.5 s after IR irradiation; an earlier time window was used as it provided better reproducibility between repeats) and the blue shading represents the initial fluorescence, to which the measurements are normalised. Bottom left: Fluorescence intensity scans of each capillary for each MST repeat prior to IR irradiation. Bottom right: Shape of the fluorescence curves of each capillary across each repeat prior to IR irradiation. Samples contained 10 mM HEPES, 50 mM NaOAc, 0.1% Tween-20, 2.5% DMSO. k_d was not measurable in this concentration range.

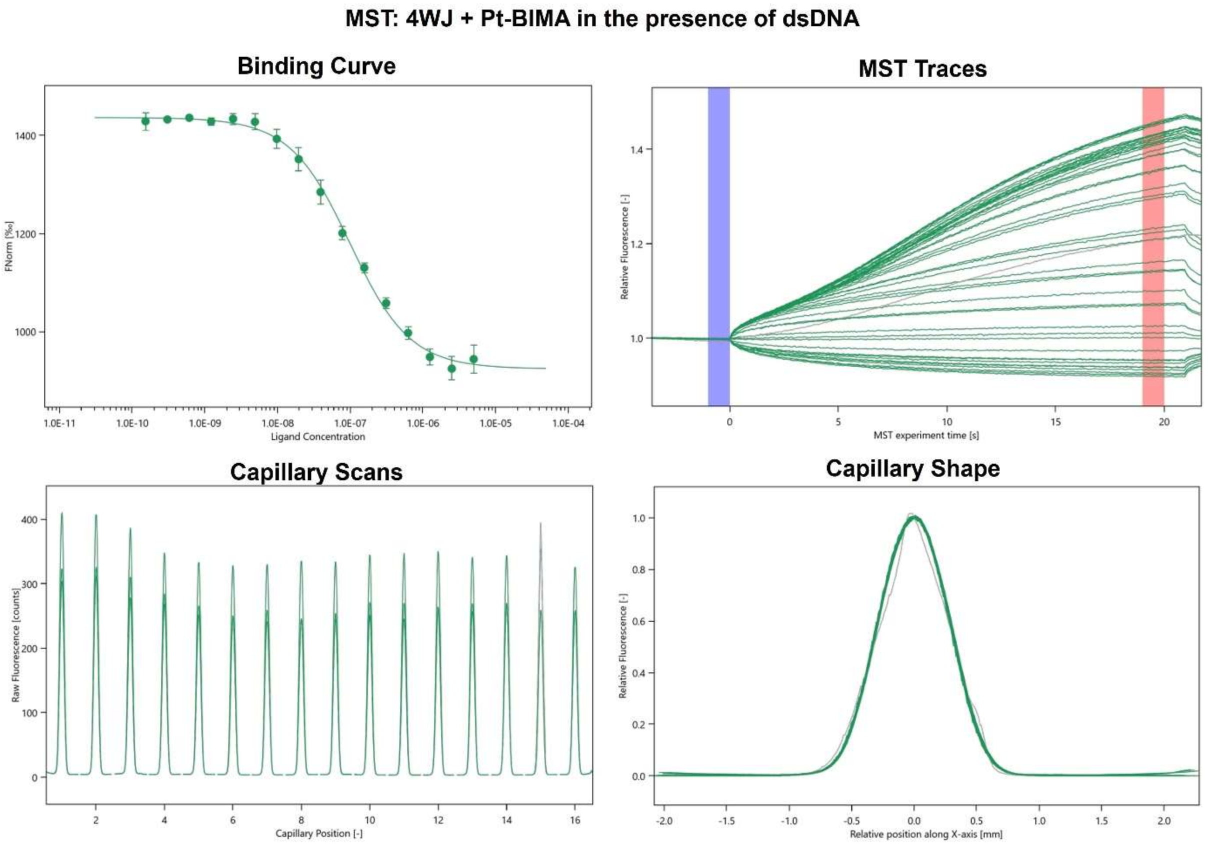

Figure S28. Top left: Binding curve obtained from MST experiments with Pt-BIMA and 4WJ in the presence of dsDNA. Pt-BIMA was serially diluted from 5 µM to 153 pM and mixed with 20 nM FAM-4WJ and 20 nM non-labelled dsDNA. Each data point is shown as the average of 3 repeats. Top right: MST traces for all samples (all repeats). The red shading represents the time range in which the average fluorescence intensity was measured to obtain the binding curve (19-20 s after IR irradiation) and the blue shading represents the initial fluorescence, to which the measurements are normalised. Bottom left: Fluorescence intensity scans of each capillary for each MST repeat prior to IR irradiation. Bottom right: Shape of the fluorescence curves of each capillary across each repeat prior to IR irradiation. The capillary corresponding to the grey curve was removed from analysis due to sample inhomogeneity. Samples contained 10 mM HEPES, 50 mM NaOAc, 0.1% Tween-20, 0.25% DMSO. k_{app} = 8.92 ± 0.63 x 10^{-8} M.

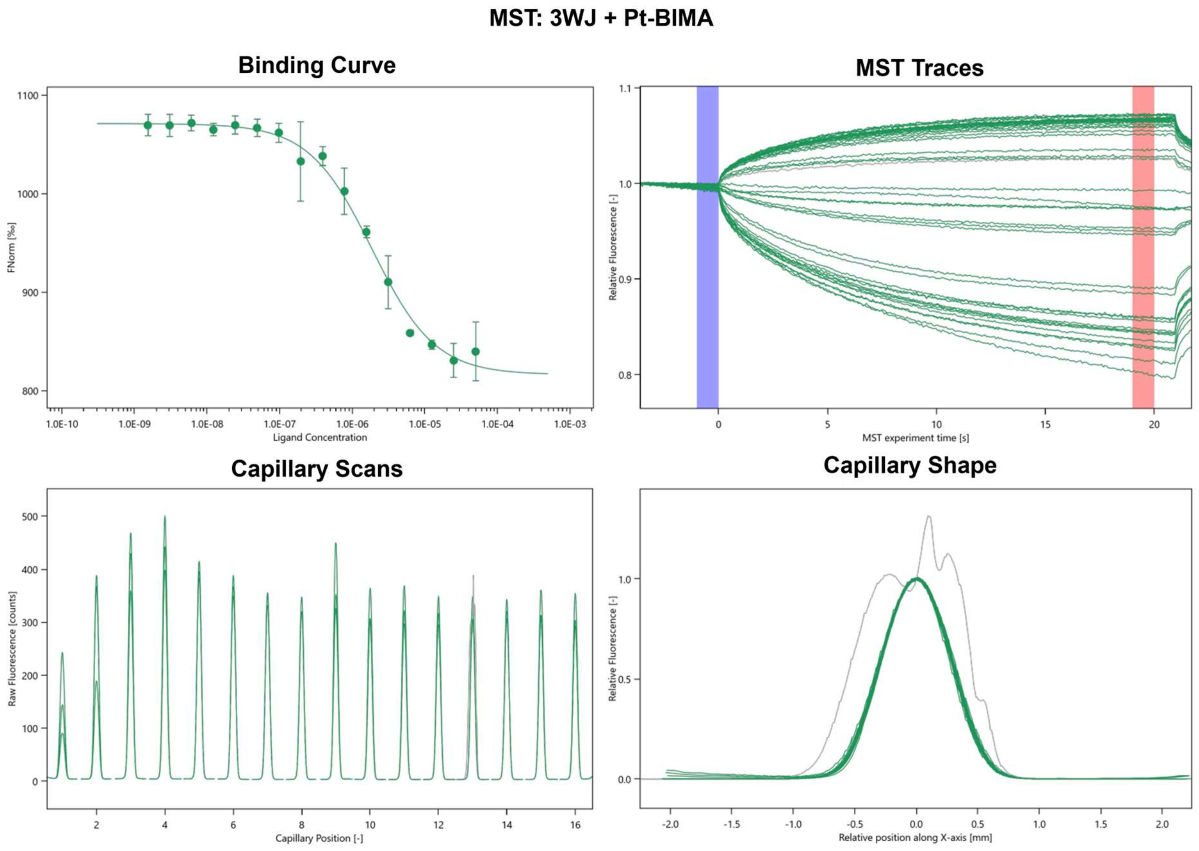

Figure S29. Top left: Binding curve obtained from MST experiments with Pt-BIMA and 3WJ. Pt-BIMA was serially diluted from 50 µM to 1.53 nM and mixed with 20 nM FAM-3WJ. Each data point is shown as the average of 3 repeats. Top right: MST traces for all samples (all repeats). The red shading represents the time range in which the average fluorescence intensity was measured to obtain the binding curve (19-20 s after IR irradiation) and the blue shading represents the initial fluorescence, to which the measurements are normalised. Bottom left: Fluorescence intensity scans of each capillary for each MST repeat prior to IR irradiation. Bottom right: Shape of the fluorescence curves of each capillary across each repeat prior to IR irradiation. The capillary corresponding to the grey curve was removed from analysis due to sample inhomogeneity. Samples contained 10 mM HEPES, 50 mM NaOAc, 0.1% Tween-20, 2.5% DMSO. $k_d = 1.90 \pm 2.27 \times 10^{-6}$ M.

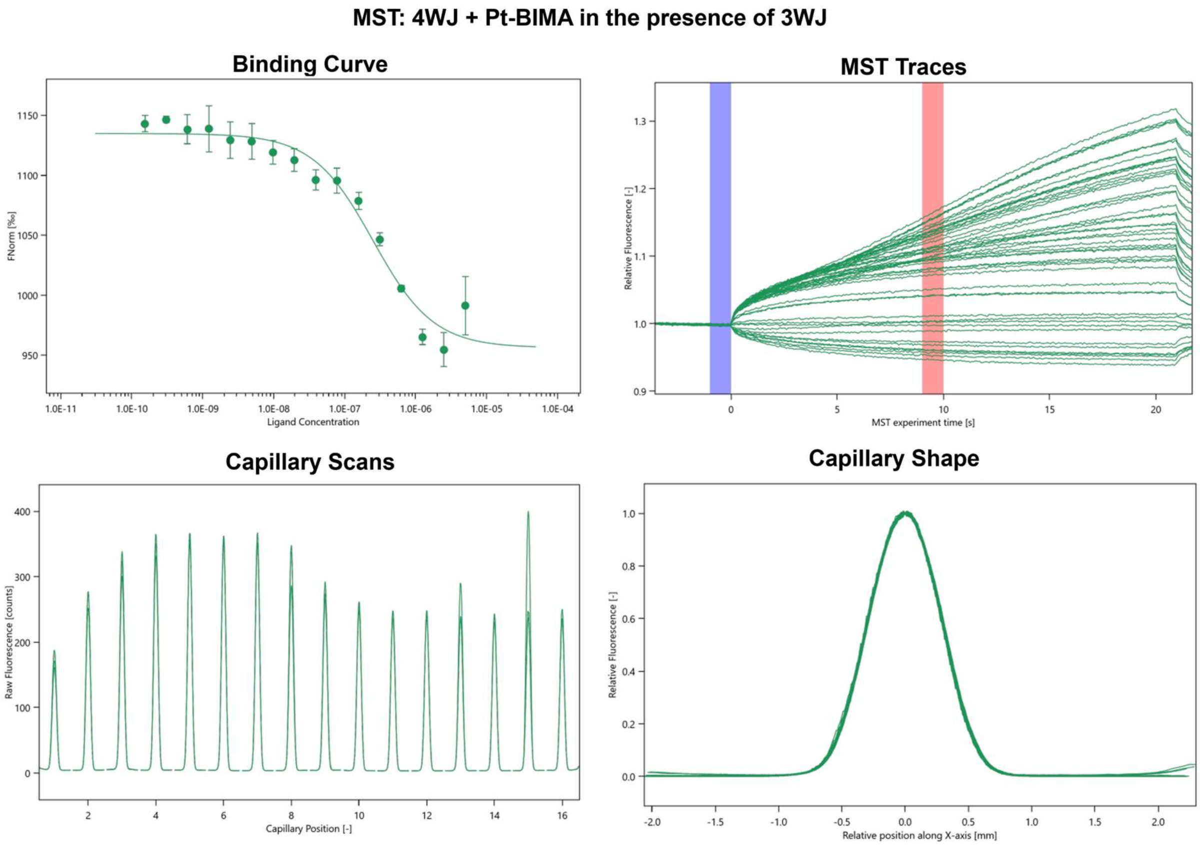

Figure S30. Top left: Binding curve obtained from MST experiments with Pt-BIMA and 4WJ in the presence of 3WJ. Pt-BIMA was serially diluted from 5 µM to 153 pM and mixed with 20 nM FAM-4WJ and 20 nM non-labelled 3WJ. Each data point is shown as the average of 3 repeats. Top right: MST traces for all samples (all repeats). The red shading represents the time range in which the average fluorescence intensity was measured to obtain the binding curve (9-10 s after IR irradiation; an earlier time window was used as it provided better reproducibility between repeats) and the blue shading represents the initial fluorescence, to which the measurements are normalised. Bottom left: Fluorescence intensity scans of each capillary for each MST repeat prior to IR irradiation. Bottom right: Shape of the fluorescence curves of each capillary across each repeat prior to IR irradiation. Samples contained 10 mM HEPES, 50 mM NaOAc, 0.1% Tween-20, 0.25% DMSO. $k_{app} = 2.41 \pm 0.64 \times 10^{-7}$ M.

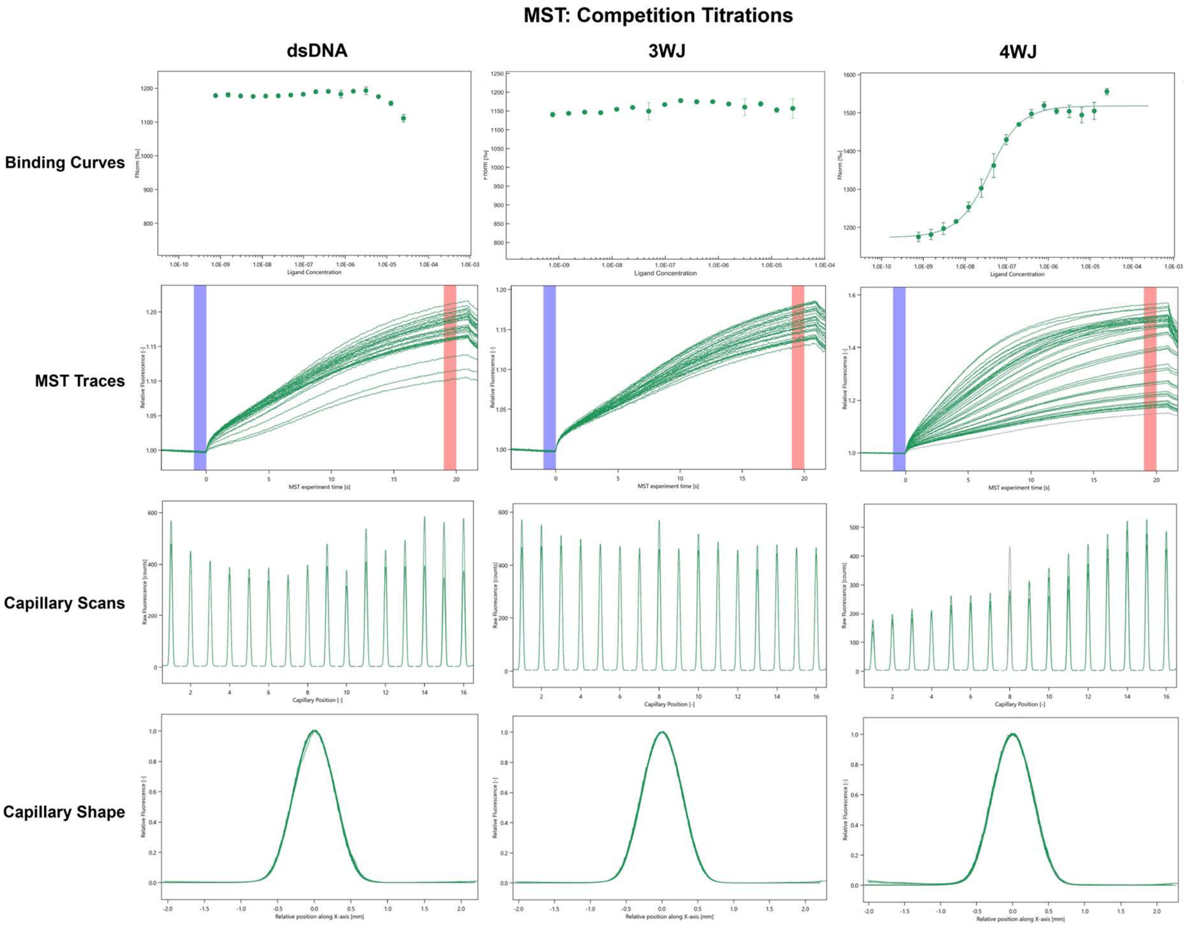

Figure S31. Binding curves (1st row), MST traces (2nd row), capillary scans (3rd row) and fluorescence curve shape (4th row) for the competition titrations. Unlabelled dsDNA (left), 3WJ (middle) and 4WJ (right) were serially diluted from 25 µM to 763 pM and mixed with 20 nM FAM-4WJ and 80 nM Pt-BIMA. Each data point in the binding curves is shown as the average of at least 2 repeats. In the MST traces, the red shading represents the time range in which the average fluorescence intensity was measured to obtain the binding curve (19-20 s after IR irradiation) and the blue shading represents the initial fluorescence, to which the measurements are normalised. Samples contained 10 mM HEPES, 50 mM NaOAc, 0.1% Tween-20, 0.25% DMSO. IC50(dsDNA) > 25 µM, IC50(3WJ) > 25 µM, IC50(4WJ) = $2.88 \pm 0.42 \times 10^{-8}$ M.

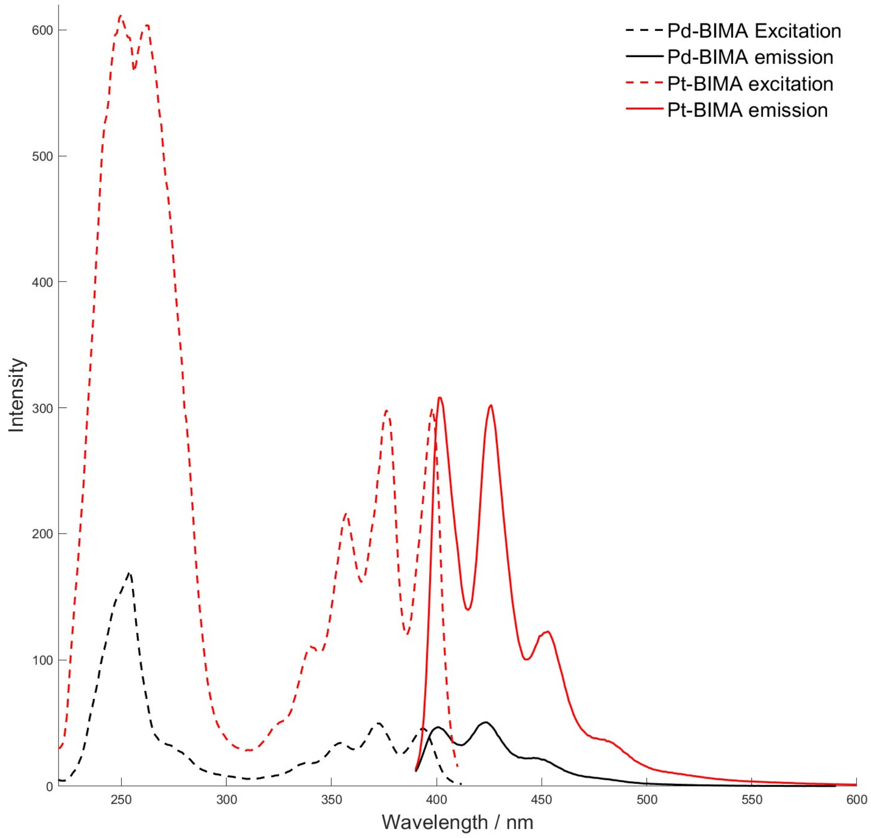

Figure S32. Fluorescence excitation and emission spectra of Pd-BIMA and Pt-BIMA in water (5µM with 0.5% or 0.25% DMSO respectively). The excitation spectrum for Pd-BIMA was recorded by monitoring the emission at 425 nm. The emission spectrum for Pd-BIMA was recorded with an excitation wavelength of 372 nm. The excitation spectrum for Pt-BIMA was recorded by monitoring the emission at 426 nm. The emission spectrum for Pt-BIMA was recorded with an excitation wavelength of 376 nm.

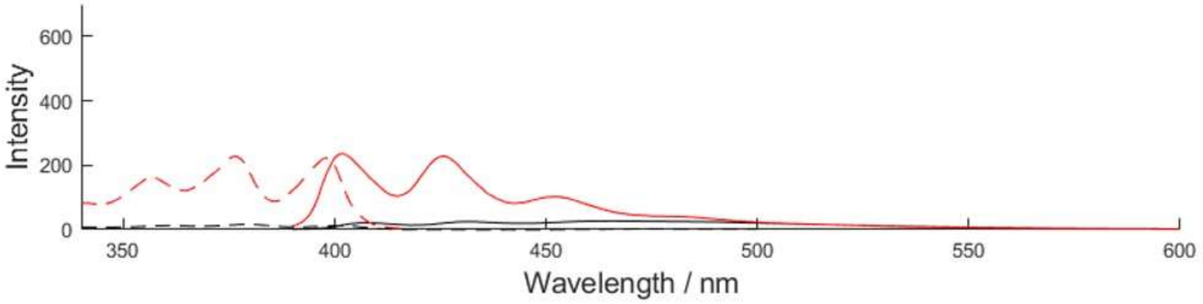

Figure S33. Fluorescence excitation and emission spectra of Pt-BIMA alone (5 µM in buffer; red lines) and Pt-BIMA with 1 equivalent non-labelled 4WJ (5 µM in buffer; black lines). The excitation spectra were recorded by monitoring the emission at 520 nm and the emission spectra were recorded with an excitation wavelength of 375 nm. The buffer used was 10 mM HEPES, 50 mM NaOAc.

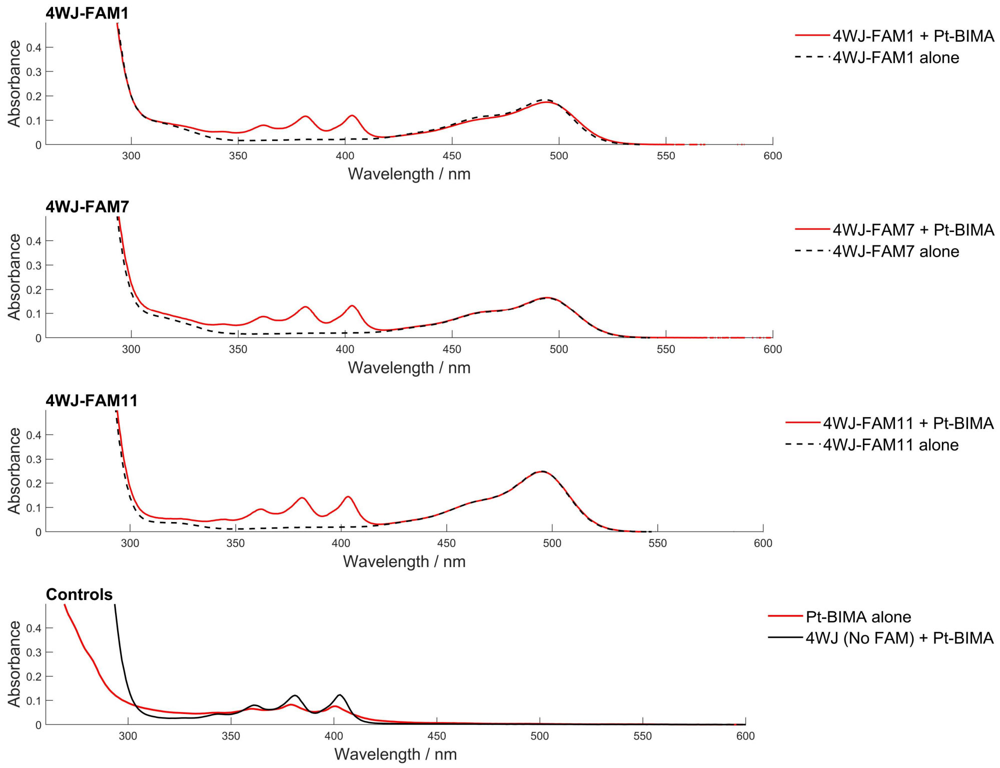

Figure S34. Absorbance spectra corresponding to the samples used in the fluorescence experiments shown in Figure 5B of the main paper (top 3) and Figure S33 (bottom).

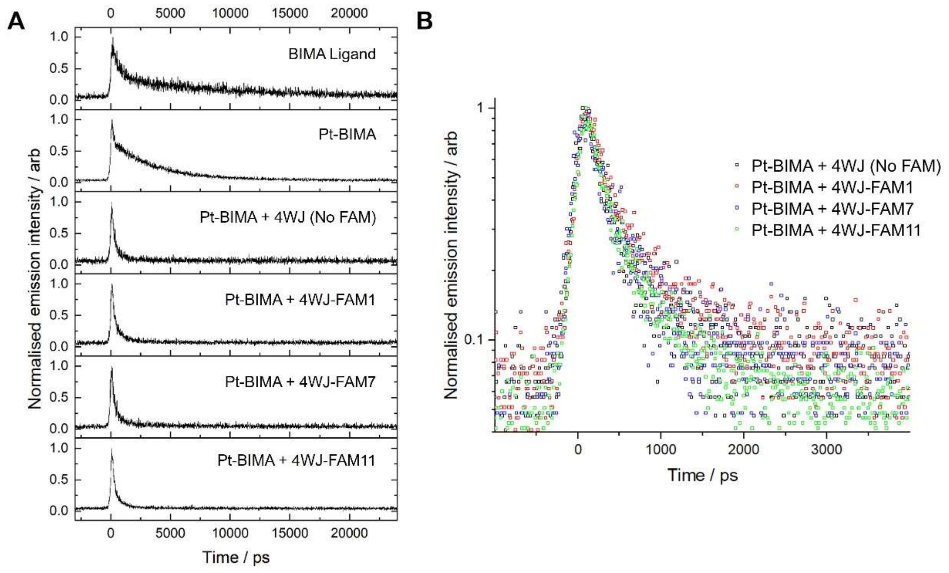

Figure S35. A) Temporal emission profiles of the BIMA ligand and Pt-BIMA, collected using TCSPC and reported over a range of environments. Emission from BIMA/Pt-BIMA is selected through the use of a 440 ± 40 nm bandpass filter to prevent contamination from FAM localised emission when present. The first two panels show a clear biexponential component to the luminescence decay, however in the presence of DNA (the lower 4 panels) the long timescale fluorescence is entirely quenched. For all four lower panels the luminescence decay has an identical lifetime within the resolution of the instrument of just over 100 ps. B) Short timescale luminescence profiles of Pt-BIMA in the presence of unlabelled 4WJ and the FAM labelled 4WJs, shown on a log scale to emphasize similarities in lifetimes. In all cases the decay profiles are identical within the uncertainties of the measurements, with an average lifetime of 110 ps.

Further Discussion on the TCSPC data

Time resolved emission profiles were recorded for the BIMA ligand, the Pt-BIMA complex, Pt-BIMA in the presence of 4WJ and also Pt-BIMA + 4WJ-FAM1, Pt-BIMA + 4WJ-FAM7 and Pt-BIMA + 4WJ-FAM11. The BIMA ligand alone exhibited biexponential behaviour, with one decay profile attributed to typical organic ligand fluorescence with a lifetime of t = 6.5(1) ns. A shorter lifetime component with a lifetime on the order of 500 ps is tentatively attributed to a ICT band which is present in this polar solvation environment. Pt-BIMA shows a similar emission profile, however upon complexation the BIMA lifetimes are reduced somewhat to roughly 100 ps for the short lifetime component and 3.41(2) ns for the long lifetime component. However, upon addition of the DNA, the long lifetime component appears entirely quenched suggesting a very strong interaction with between the Pt-BIMA and the DNA. Addition of the FAM tags generates new emission bands, which can be wavelength separated from the complex emission bands. The ligand/complex emission bands can continue to be examined using a 440 ± 40 nm bandpass filter (although this somewhat reduces the signal to noise of these measurements), with emission from the FAM tag examined using a 500 nm long pass filter. The emission profile of the ligand in these varying environments can be seen in Figure S35.

Figure S35A demonstrates that in the presence of the DNA with any FAM tag position (or indeed no FAM), the longer timescale fluorescence of the complex is entirely quenched, with only the short timescale component remaining, albeit reduced in intensity. Given the magnitude of this quenching it is not possible to resolve changes in the magnitude of the complex quenching based upon the position of the FAM within the DNA backbone, although the counts per shot are certainly lowest for the Pt-BIMA + 4WJ-FAM1 system. Lifetimes of the short timescale component of the complex emission are approaching the resolution of the instrument, and within uncertainty the lifetimes are all comparable at t = 110 ps (*Fig. S35B*), for example where Pt-BIMA FAM1 gives a lifetime of τ = 119(11) ps and FAM11 of τ = 109(6) ps. Thus, confirmation of FRET in the 4W-FAM systems based on the donor lifetime was not possible. Additionally, there are some small differences observable in the lifetimes of the FAM emission, where FAM1 shows the longest lifetime of t = 3932(15) ps, with FAM7 and FAM11 providing lifetimes of t = 3593(25) ps and 3520(15) ps respectively. This change in lifetime may reflect the slightly different environments experienced by the FAM tag in each position.

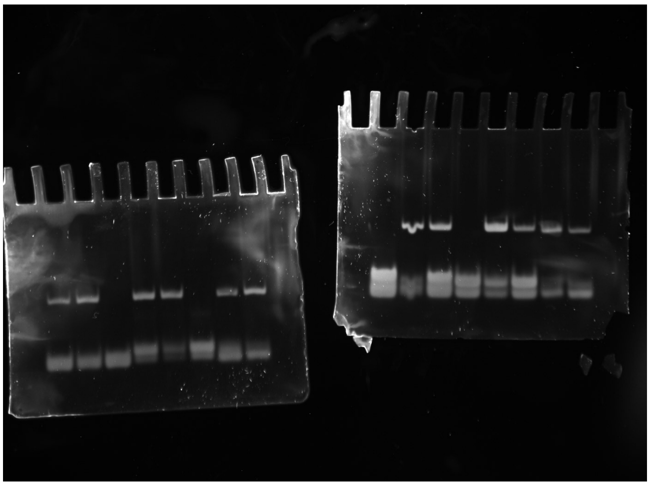

Figure S36. Raw image of the two gels shown in Figure 6C (left) and Figure 6D (right) of the main paper. The two gels were run in the same tank and running buffer, in parallel, simultaneously and for the same period of time, at the same electrical potential and then stained and imaged together. The gel on the left contains 3WJ with Pd-BIMA and Pt-BIMA (lanes 1-2), a 3WJ sequence not discussed in this paper (lanes 3-5) and the mismatched 3WJ alone, and with Pd-BIMA and Pt-BIMA (lanes 6-8). The middle 3 lanes were removed for Figure 6C as this data was not discussed in this paper. The gel on the right contains the one-base-bulged 3WJ alone and with Pd-BIMA and Pt-BIMA (lanes 1-3), the two-base-bulged 3WJ alone and with Pd-BIMA and Pt-BIMA, and 3WJ with Pd-BIMA and Pt-BIMA. The inclusion of the control lanes (3WJ + Pd and Pt-BIMA; aliquots of the same samples were loaded onto both gels) on both gels allowed for direct comparison of the gel shifts between the two gels.

REFERENCES

[1] M. J. Hannon, C. L. Painting, A. Jackson, J. Hamblin, W. Errington, *ChemComm.* **1997**, 1807-1808.

[2] P. J. Altmann, A. Pöthig, *J. Am. Chem. Soc.* **2016**, *138*, 13171-13174.

[3] R. W. Sabnis, D. J. Guerrero, T. Brewer, M. J. Spencer, US/2001/0021481, **2001**.

[4] C. Yu, X. Wang, T. Wu, X. Gu, W. Huang, A. M. Kirillov, D. Wu, *Dalton Trans.* **2020**, *49*, 12082-12087.

[5] L.-P. Zhou, Q.-F. Sun, *ChemComm.* **2015**, *51*, 16767-16770.

[6] J. H. Price, A. N. Williamson, R. F. Schramm, B. B. Wayland, *Inorg. Chem.* **1972**, *11*, 1280-1284.

[7] C. A. Schneider, W. S. Rasband, K. W. Eliceiri, *Nat. Methods* **2012**, *9*, 671-675.

[8] B. McGorman, S. Poole, M. V. López, A. Kellett, *Methods* **2023**, *219*, 30-38.

[9] D. A. Smith, G. McKenzie, A. C. Jones, T. A. Smith, *Methods Appl. Fluoresc.* **2017**, *5*, 042001.

[10] B. Ambrose, J. M. Baxter, J. Cully, M. Willmott, E. M. Steele, B. C. Bateman, M. L. Martin-Fernandez, A. Cadby, J. Shewring, M. Aaldering, T. D. Craggs, *Nat. Commun.* **2020**, *11*, 5641.

[11] M. J. Frisch, G. W. Trucks, H. B. Schlegel, G. E. Scuseria, M. A. Robb, J. R. Cheeseman, G. Scalmani, V. Barone, G. A. Petersson, H. Nakatsuji, X. Li, M. Caricato, A. V. Marenich, J. Bloino, B. G. Janesko, R. Gomperts, B. Mennucci, H. P. Hratchian, J. V. Ortiz, A. F. Izmaylov, J. L. Sonnenberg, Williams, F. Ding, F. Lipparini, F. Egidi, J. Goings, B. Peng, A. Petrone, T. Henderson, D. Ranasinghe, V. G. Zakrzewski, J. Gao, N. Rega, G. Zheng, W. Liang, M. Hada, M. Ehara, K. Toyota, R. Fukuda, J. Hasegawa, M. Ishida, T. Nakajima, Y. Honda, O. Kitao, H. Nakai, T. Vreven, K. Throssell, J. A. Montgomery Jr., J. E. Peralta, F. Ogliaro, M. J. Bearpark, J. J. Heyd, E. N. Brothers, K. N. Kudin, V. N. Staroverov, T. A. Keith, R. Kobayashi, J. Normand, K. Raghavachari, A. P. Rendell, J. C. Burant, S. S. Iyengar, J. Tomasi, M. Cossi, J. M. Millam, M. Klene, C. Adamo, R. Cammi, J. W. Ochterski, R. L. Martin, K. Morokuma, O. Farkas, J. B. Foresman, D. J. Fox, Wallingford, CT, **2016**.

[12] P. Li, K. M. Merz, Jr., *J. Chem. Inf. Model.* **2016**, *56*, 599-604.

[13] M. D. Hanwell, D. E. Curtis, D. C. Lonie, T. Vandermeersch, E. Zurek, G. R. Hutchison, *J. Cheminform.* **2012**, *4*, 17.

[14] D. A. Case, H. M. Aktulga, K. Belfon, D. S. Cerutti, G. A. Cisneros, V. W. D. Cruzeiro, N. Forouzesh, T. J. Giese, A. W. Götz, H. Gohlke, S. Izadi, K. Kasavajhala, M. C. Kaymak, E. King, T. Kurtzman, T.-S. Lee, P. Li, J. Liu, T. Luchko, R. Luo, M. Manathunga, M. R. Machado, H. M. Nguyen, K. A. O'Hearn, A. V. Onufriev, F. Pan, S. Pantano, R. Qi, A. Rahnamoun, A. Risheh, S. Schott-Verdugo, A. Shajan, J. Swails, J. Wang, H. Wei, X. Wu, Y. Wu, S. Zhang, S. Zhao, Q. Zhu, T. E. Cheatham, III, D. R. Roe, A. Roitberg, C. Simmerling, D. M. York, M. C. Nagan, K. M. Merz, Jr., *J. Chem. Inf. Model.* **2023**, *63*, 6183-6191.

[15] K. C. Woods, S. S. Martin, V. C. Chu, E. P. Baldwin, *J. Mol. Biol.* **2001**, *313*, 49-69.

[16] J. S. Craig, L. Melidis, H. D. Williams, S. J. Dettmer, A. A. Heidecker, P. J. Altmann, S. Guan, C. Campbell, D. F. Browning, R. K. O. Sigel, S. Johannsen, R. T. Egan, B. Aikman, A. Casini, A. Pöthig, M. J. Hannon, *J. Am. Chem. Soc.* **2023**, *145*, 13570-13580.

[17] K. Ghosh, C. K. Lau, F. Guo, A. M. Segall, G. D. Van Duyne, *J. Biol. Chem.* **2005**, *280*, 8290-8299.

[18] I. Ivani, P. D. Dans, A. Noy, A. Pérez, I. Faustino, A. Hospital, J. Walther, P. Andrio, R. Goñi, A. Balaceanu, G. Portella, F. Battistini, J. L. Gelpí, C. González, M. Vendruscolo, C. A. Laughton, S. A. Harris, D. A. Case, M. Orozco, *Nat. Methods* **2016**, *13*, 55-58.

[19] M. J. Abraham, T. Murtola, R. Schulz, S. Páll, J. C. Smith, B. Hess, E. Lindahl, *SoftwareX* **2015**, *1-2*, 19-25.

[20] L. Melidis, I. B. Styles, M. J. Hannon, *Chem. Sci.* **2021**, *12*, 7174-7184.

[21] The PyMOL Molecular Graphics System, Version 2.8, Schrödinger, LLC.
